# Using sensory and instrumental analysis to assess the impact of grape smoke exposure on different red wine varietals in California

**DOI:** 10.1038/s41598-024-77041-1

**Published:** 2024-11-07

**Authors:** Lik Xian Lim, Cristina Medina-Plaza, Ignacio Arías-Perez, Yan Wen, Bishnu Neupane, Larry Lerno, Jean-Xavier Guinard, Anita Oberholster

**Affiliations:** 1 Department of Viticulture and Enology, University of California Davis, Davis, CA 95616 USA; 2 Department of Food Science and Technology, University of California Davis, Davis, CA 95616 USA

**Keywords:** Smoke taint, Wine quality, Sensory evaluation, Flavor chemistry, Environmental sciences, Environmental chemistry, Environmental impact, Analytical chemistry

## Abstract

This study is an investigation of the impact of volatile phenols (VPs) released from burning wood during wildfires on grape composition and the resulting wines. Baseline levels of VPs in grapes and sensory differences between smoke-impacted wines and non-smoke-impacted wines were determined. The differences were related to different levels of smoke taint marker compounds in different wine matrices, using modified descriptive analysis (DA), multivariate statistics, gas chromatography mass spectrometry (GC-MS) and liquid chromatography-triple quadrupole tandem mass spectrometry (LC-QqQ-MS) of the free and total VPs, and individual bound glycosides, respectively. Across two DA panels, Cabernet Sauvignon, Cabernet Franc, Petite Verdot, Merlot, Syrah, Malbec, and Zinfandel wines made from grape originating from different areas in California were evaluated. The results show sensory differences between highly smoke-impacted and non-impacted wines with wines made from highly smoke-impacted grapes characterized as smoky, barbeque, medicinal, and having a retro-nasal ashtray character. Low smoke-impact wines based on free and total VP concentrations were not significantly different from the non-impacted wines when rated through descriptive analysis. The amount of smoke exposure was the largest contributor to smoke impact determined by sensory evaluation, but the different wine matrices from different locations and varietals also played an important role in determining the level of perceived smoke impact. The results of this study will contribute to our understanding of smoke impact and how it influences wine characteristics by relating smoke marker indicator compounds to wine sensory attributes.

**Supplementary Information:**

The online version contains supplementary material available at 10.1038/s41598-024-77041-1.

## Introduction

Wildfires in California have become more frequent and destructive in recent years with some of the largest fires in California such as the Nuns fire and Atlas fires taking place in 2017, and LNU lightning complex and Grass fires which occurred in 2020^1–3^. When wildfires occur, large amounts of pollutants are released into the air, including ash, gaseous pollutants, and other volatile organic compounds, such as volatile phenols (VPs)^4^. These VPs are absorbed through the grape berry skin, where they are quickly glycosylated^5^. The wines made from smoke-exposed grapes are often classified as smoke-impacted due to their smoky, burnt, ashy, barbeque, medicinal aromas, and retro nasal ashtray aftertaste^6–10^. Wines of such nature are smoke-impacted and are unsalable. The result is a large economic loss for the California wine industry, particularly in Napa Valley, in fire-affected years.

There is a family of VP marker compounds, guaiacol, *o-*cresol, *p-*cresol, *m-*cresol, 4-methyl-guaiacol, syringol, and 4-ethylguaiacol as identified by Parker et al.^5,11^. These VPs exist in both free and bound forms where the free VP binds with compounds such as sugars to form phenolic glycosides^5,12,13^. The presence of the ashy aftertaste can be attributed to the breakdown of the phenolic glycosides due to enzymes in the saliva^14^. Free VPs can be released from the bound during various winemaking stages such as grape processing, fermentation, and bottle aging^14–19^. This contributes to the amount of free VPs present which would be perceived as “ashy” retronasally. Current research has shown that despite being able to measure the important smoke marker compounds, it is difficult to accurately predict smoke impact in a particular wine matrix due to large variations in their baseline levels in grapes. VP biosynthesis will likely be influenced by the grape variety, and locations where is it grown^6^.

Wine is a complex matrix made up of water, alcohol, sugars, organic acids, amino acids, phenols, minerals, and many other compounds. All these compounds work synergistically to make a wine that is unique. Wines produced from different varieties of grapes will have different proportions of these compounds, leading to matrix differences. Similarly, wines made in different regions from the same grape variety would also have a different matrix due to the myriad of effects combining location, climate, environmental factors, and farming practices. Importantly, when understanding smoke, there may be underlying volatile phenols already present that are unique to the varietal and to the general region it is from^16,20,21^.

Descriptive analysis can be used as a tool to determine the level of smoke impact^22,23^. However, traditional descriptive analysis requires long training sessions and a dedicated panel of judges who are trained through a consensus methodology on specific attributes of wine to be able to discriminate them from each other. This is non-optimal for the industry as there is little time afforded for training, nor excess resources required for the evaluations during a short growing season. Rapid methods of descriptive analysis such as rate-all-that-apply (RATA), Flash Profile, and check-all-that-apply (CATA) have been widely adopted in the modern context as they are faster, require less resources and give similar results to classical descriptive analysis^24–27^.

The objective of the study was to determine the impact of smoke exposure on the sensory quality of different wine matrices as a function of variety, location, and level of smoke exposure.

## Materials and methods

### Grapes

During the smoke event (LNU Lightning complex fire and Grass wildfires) that occurred in the 2020 harvest in California, grapes naturally exposed to the smoke were harvested. Smoke exposure is usually measured using air sampling at the vineyard of concern. However, due to the nature of the fire event, we were not able to access the vineyard sites at the time of the event to do air sampling. Hence, Air Quality Index (AQI) was used to determine smoke exposure. In total, there were nine Cabernet Sauvignon wines from nine different sites and six commonly found red wine varieties: Cabernet Franc, Petite Verdot, Merlot, Syrah, Malbec, and Zinfandel from across California’s North Coast grape growing region. In 2021, non-impacted fruit from the same vineyard for some of the varieties were harvested to make a non-impacted control (Table 1). Also in 2021, a single lot of Cabernet Sauvignon was split into two equal lots where one lot was intentionally smoked on drying tables in a purpose-built smoking tent covered with six-mil polyethene sheeting (Frost King & Thermwell Products Co., Inc., Mahwah, NJ, USA). The grapes were smoked for one hour using a Z Grills pellet smoker (Z Grills Inc., Ontario, CA, USA) with 100 g of hickory wood pellets from Traeger (Traeger, Salt Lake City, UT, USA) to give a wine of similar matrix but with maximum smoke impact^8^. Hickory pellets was used as it gave a very similar VPs profile when compared to the VPs released from the ash collected in the 2020 natural fire event (data not shown). All grapes were collected with permission from commercially farmed vineyards.

**Table 1:** Wine coding scheme with year, varietal, AVA, county, smoke exposure status, and smoke exposure period where AQI > 150.

Wine name	Year	Varietal	AVA	County	Taint	Smoke exposure period	DA Panel
20CS_A_ST	2020	Cabernet Sauvignon	Napa Valley	Napa	ST	19 Aug 2020–21 Sep 2020 (5 days > 150); 17 Sep 2020–5 Oct 2020 (2 days > 150)	1
20CS_B_ST	2020	Cabernet Sauvignon	St. Helena	Napa	ST	19 Aug 2020–21 Sep 2020 (5 days > 150); 17 Sep 2020–5 Oct 2020 (2 days > 150)	1
20CS_C_ST	2020	Cabernet Sauvignon	St. Helena	Napa	ST	19 Aug 2020–21 Sep 2020 (5 days > 150); 17 Sep 2020–5 Oct 2020 (2 days > 150)	1
20CS_D_ST	2020	Cabernet Sauvignon	Russian River Valley	Sonoma	ST	19 Aug 2020–21 Sep 2020 (5 days > 150)	1
20CS_E_ST	2020	Cabernet Sauvignon	Dry Creek Valley	Sonoma	ST	19 Aug 2020–21 Sep 2020 (5 days > 150	1
20CS_F_ST	2020	Cabernet Sauvignon	Oakville	Napa	ST	19 Aug 2020–21 Sep 2020 (5 days > 150); 17 Sep 2020–5 Oct 2020 (2 days > 150)	1
20CS_G_ST	2020	Cabernet Sauvignon	Spring Mountain District	Napa	ST	19 Aug 2020–21 Sep 2020 (5 days > 150); 17 Sep 2020–5 Oct 2020 (2 days > 150)	1
20CS_H_ST	2020	Cabernet Sauvignon	Davis (No AVA)	Yolo	ST	19 Aug 2020–21 Sep 2020 (5 days > 150); 17 Sep 2020–8 Oct 2020 (3 days > 150)	1
21CS_I_NST	2021	Cabernet Sauvignon	Dry Creek Valley	Sonoma	NST	16 Aug 2021–21 Sep 2021 (2 days > 150)	1
21CS_J_NST	2021	Cabernet Sauvignon	Oakville	Napa	NST	16 Aug 2021- 21 Sep 2021 (1 days > 150)	1
21CS_K_ST	2021	Cabernet Sauvignon	Oakville	Napa	ST	Intentional smoking	1
20CF_A_ST	2020	Cabernet Franc	Napa Valley	Napa	ST	19 Aug 2020–21 Sep 2020 (5 days > 150); 17 Sep 2020–5 Oct 2020 (2 days > 150)	2
20CF_B_ST	2020	Cabernet Franc	St. Helena	Napa	ST	19 Aug 2020–21 Sep 2020 (5 days > 150); 17 Sep 2020–5 Oct 2020 (2 days > 150)	2
20CF_C_ST	2020	Cabernet Franc	Oakville	Napa	ST	19 Aug 2020–21 Sep 2020 (5 days > 150); 17 Sep 2020–5 Oct 2020 (2 days > 150)	2
20MA_D_ST	2020	Malbec	Dry Creek Valley	Sonoma	ST	19 Aug 2020–21 Sep 2020 (5 days > 150)	2
20ME_E_ST	2020	Merlot	Oakville	Napa	ST	19 Aug 2020–21 Sep 2020 (5 days > 150); 17 Sep 2020–5 Oct 2020 (2 days > 150)	2
20ME_F_ST	2020	Merlot	Spring Mountain District	Napa	ST	19 Aug 2020–21 Sep 2020 (5 days > 150); 17 Sep 2020–5 Oct 2020 (2 days > 150)	2
20PV_G_ST	2020	Petite Verdot	Napa Valley	Napa	ST	19 Aug 2020–21 Sep 2020 (5 days > 150); 17 Sep 2020–5 Oct 2020 (2 days > 150)	2
20PV_H_ST	2020	Petite Verdot	St. Helena	Napa	ST	19 Aug 2020–21 Sep 2020 (5 days > 150); 17 Sep 2020–5 Oct 2020 (2 days > 150)	2
20SY_I_ST	2020	Syrah	Dry Creek Valley	Sonoma	ST	19 Aug 2020–21 Sep 2020 (5 days > 150)	2
20ZN_J_ST	2020	Zinfandel	Davis (No AVA)	Yolo	ST	19 Aug 2020- 21 Sep 2020 (5 days > 150); 17 Sep 2020–8 Oct 2020 (3 days > 150)	2
21MA_K_NST	2021	Malbec	Dry Creek Valley	Sonoma	NST	16 Aug 2021–21 Sep 2021 (2 days > 150)	2
21SY_L_NST	2021	Syrah	Dry Creek Valley	Sonoma	NST	16 Aug 2021–21 Sep 2021 (2 days > 150)	2

*AQI- Air Quality Index, AVA- American Viticultural Areas.

### Winemaking

Grapes were hand harvested in 2020 and 2021 from the different vineyards in California (USA) and transported to the UC Davis Teaching and Research Winery (Davis, CA, USA) for processing. Grapes, on average 120 kg per fermentation replicate, were destemmed and crushed using a Bucher Vaslin Delta E2 (Bucher Vaslin North America, Santa Rosa, CA, USA) into stainless steel vessels. 50 mg/L of sulfur dioxide (SO2) was added using a 15% potassium metabisulfite solution (Laffort, Petaluma, CA, USA). Additions were made to each vessel to adjust yeast assimilable nitrogen (YAN) to 250 mg/L, if needed, using diammonium phosphate (Laffort, Petaluma, CA, USA), and titratable acidity (TA) to 6.0 g/L, if needed, using tartaric acid (CalSoda, Rohnert Park, CA, USA). Fermentations were carried out using *Saccharomyces cerevisiae* strain EC1118 (Lallemand, Montreal, Canada) and inoculated according to the rehydration procedure described by the manufacturer. Fermentation temperature was controlled at 25 °C, while cap management conditions were set to one tank volume pump-over twice a day. After seven days of maceration, wines were pressed and inoculated with *Oenococcus oeni* VP41 to induce malolactic fermentation (MLF) (Lallemand, Montreal, Canada). MLF was considered completed when malic acid levels were under 0.2 g/L (approximately 4 weeks). Prior to bottling, all vessels of each wine were blended after evaluation for similarity and wine faults other than smoke and adjusted to 35 mg/L free SO_2_ using potassium metabisulfite (Laffort, Petaluma, CA, USA). Wines were rough filtered via plate and frame unit using FibraFix AF 100 depth filter sheets (Filtrox, St. Gallen, Switzerland). Subsequently, wines were sterile filtered using in-line ALpHA MF0.8-1F6RS and SteriLUX VMH0.4-1F6RS filters (Meissner, Camarillo, CA) and then bottled in antique green bottles under screw cap (Saranex liner. Amcor, Zurich, Switzerland) and stored at 14 °C until analysis.

### Chemical analysis

#### Free and acid-labile (total) volatile phenols

The guaiacol, creosol (4-methylguaiacol), *o*-cresol, phenol, 4-ethylguaiacol, *p*-cresol, *m*-cresol, 2,3-dimethoxyphenol, 4-ethylphenol, syringol and 4-methylsyringol in samples was quantified using a liquid-liquid extraction with pentane-ethyl acetate (1:1) as in Oberholster et al.^18^. Thereafter an Agilent 7890A gas chromatograph coupled to an Agilent 7000B triple quadrupole mass spectrometer with an MPS 2 autosampler (Gerstel, Inc., Linthicum, MD) was used with the following conditions. A DB-WAXetr (30 m length × 0.32 mm i.d. × 1.0 μm film thickness, Agilent Technologies, Santa Clara, CA, USA) column was fitted onto the gas chromatogram. The inlet temperature was held constant at 220 °C. Oven program was held at 75 °C for 1 min initially, increased to 180 °C at a rate of 15 °C/min, increased to 230 °C at a rate of 10 °C/min and held for another 1 min. Finally, temperature was increased to 250 °C at a rate of 50 °C/min and held for 3 min. Total run time was 17.4 min. The GC and MS interface was held at 220 °C. Pulsed splitless mode was used. The split vent was opened at 1 min with a flow of 50 mL/min. Helium carrier gas was used in constant flow mode at 2.0 mL/min and the electron ionization source set at 70 eV.

The source was held at 230 °C and reagent gas, helium was introduced to the source at 1 mL/min. The solvent delay was 7.5 min. Multiple reaction monitoring (MRM) quantitative and qualitative transitions and collision energies were chosen for each compound based on signal-to-noise ratios using commercially available standards (Supplementary Table S1). The dwell times were set with 15 scans over each peak to ensure quantitative peak integration. The helium quench gas and nitrogen collision gas were fixed at 2.25 mL/min and 1.5 mL/min, respectively.

#### Phenolic glycoside analysis

The concentrations of smoke glycosides were determined using a solid phase extraction (SPE) SPE extraction and were analyzed by liquid chromatography tandem mass spectrometry (LC-MS/MS) in accordance with the method used in Oberholster et al.^18^. Liquid chromatography was performed on an Agilent 1290 Infinity UHPLC (Agilent Technologies, Santa Clara, CA) equipped with a binary pump, temperature controlled autosampler, and a thermostated column compartment. The column employed for chromatographic separation was an Agilent Poroshell Bonus-RP (150 mm × 2.1 mm, 2.7 μm) fitted with a matching guard column and maintained at 40 °C. Mobile phase A was water with 10 mM ammonium formate and mobile phase B was methanol: acetonitrile (1:1) with 10 mM ammonium formate. The flow rate of the mobile phase was 0.42 mL/min. The gradient used for the separation was as follows: 0 min, 8% B; 1 min, 8% B; 6.5 min, 24.5% B; 7.5 min, 90% B; 9 min, 90% B; 10 min, 8% B. The column was equilibrated at starting conditions for two minutes before the next injection. The injection volume was 12 µL for all samples.

Tandem mass spectrometry was performed on an Agilent 6460 triple quadrupole mass spectrometer (Agilent Technologies, Santa Clara, CA) with an Agilent JetStream electrospray source. Source conditions were sheath gas temperature 375 °C, sheath gas flow 11 L/min, drying gas temperature 250 °C, drying gas flow 12 L/min, nebulizer pressure 45 psi, capillary voltage 3500 V, and nozzle voltage 0 V. Detection of the glycosides was done using dynamic MRM. MRM transitions were determined and optimized using commercially available standards (Supplementary Table S2). Analytical grade chemicals and HPLC grade solvents were purchased from Sigma-Aldrich and Merck (Darmstadt, Germany). Calibration curves were constructed for all glycosides. Deuterated VP glycosides were used as internal standards. Deuterated standards were obtained from Toronto Research Chemicals (Toronto, Canada), C/D/N Isotopes Inc. (Quebec, Canada) and EPTES (Vevey, Switzerland).

#### Wine analysis

The chemical parameters of the wines were analyzed on each testing day of the descriptive analysis. The titratable acidity (TA) was measured using a Mettler-Toledo DL50 titrator (Mettler- Toledo Inc., Columbus, OH, USA); pH was measured using an Orion 5-star pH meter (Thermo Fisher Scientific, Waltham, MA, USA); alcohol content % (v/v) was measured using an alcohol analyzer (Anton Parr, Ashland, VA, USA); acetic acid, malic acid and residual sugar (RS) were determined by enzymatic analysis using the Gallery automated analyzer (Thermo Fisher Scientific, Waltham, MA, USA).

### Sensory analysis

#### Panel recruitment

Panelists were recruited from an in-house panel. They were initially recruited based on interest, availability, and consumption frequency of red wine (at least once a week). All panelists were screened for their ability to detect smoke using difference tests for the ashy standard diluted to varying concentrations. Panelists were all experienced at tasting red wines and in descriptive analysis (DA).

The study and recruitment were conducted in accordance to the guidelines of the Declaration of Helsinki with approval from the Institutional Review Board (IRB) of the University of California, Davis—UC Davis IRB Protocol 1288072-1. Informed consent was obtained from all participants in the study.

#### Modified descriptive analysis

A modified descriptive analysis method was used^24^. Wines were first bench-tasted by three sensory panel leaders, who generated descriptors along with reference standards. This list of descriptors and a set of reference standards were provided to the panel on the first day of training. The panel came to a consensus that standards and descriptors were sufficient in describing the set of wines^28^. There was a total of fifteen aroma standards, six mouthfeel/taste standards, and one ashy standard (Supplementary Table, S3). Amongst the fifteen aroma standards, seven of them were smoke-related attributes. Panelists underwent five training sessions which included familiarization with the system used, Redjade (Redjade, Redwood City, CA, USA)). All panelists were additionally screened for performance during the training sessions where underperforming panelists had additional training. Panelists were all eligible to participate in both DA panels, DA1 (*n* = 15), and DA2 (*n* = 14), with the completed training. One panelist did not complete DA2 and one panelist was dropped in each panel due to poor panel performance (i.e., inconsistency between replicates) for a final count of 14 panelists in DA1 (7 male, 7 female, ages 22–64 years old) and 13 panelists in DA2 (6 male, 7 female, ages 22–64 years old).

Training and evaluations took place in April 2022 where the DA panels DA1 and DA2 ran one after another. There was a total of six days of evaluations for each of the DA panels. Panelists evaluated six wines each day for both DA panels. Formal evaluations were held in positive pressured red-light booths, with 30 ml of wine served in a black Riedel wine glass, item number #0446 Zinfandel/Riesling (Riedel Crystal of America, Edison, NJ, USA). Each day, participants had to complete an aroma quiz before participating in the evaluations. Participants were given a code to log in to Redjade where they were prompted with a 3-digit binding code assigned to each wine. All wines were tasted in triplicate, in a randomized block design. Panelists were to first assess the aroma of the wine without tasting the wine, then to take a sip of the wine, expectorate, and evaluate the taste and mouthfeel attributes. Finally, they would take another sip and evaluate the ashy retronasal aftertaste over a thirty-second period after expectoration where they would rate the highest level of the retro nasal ashy character. The participants rated each attribute on a 15 cm unstructured line scale, with anchors at 0% (not present), 10% (low presence), 90% (very intense), 100% (max intensity). There was an enforced 2-min wait between samples to minimize carryover effects^18,29^. Panelist were instructed to first use the provided dextrose solution (4 g/L) to rinse their mouths between samples. Crackers and plain still mineral water were available for the panelists as well.

#### Ashy standard

The ashy standard was prepared as described in Fryer et al.^9^ with the following changes. The burnt leeks tips were crushed and mixed with 100 °C hot water (Crystal Geyser Natural Alpine Spring Water, Novato, CA, USA) for a 10% weight-by-volume solution. It was allowed to sit in a Abid clever coffee dripper (Abid Co., Ltd, Taiwan) with a coffee filter (Melitta North America, Inc, Florida, USA), for two hours at room temperature. The 10% w/v solution was filtered and diluted at a 1:5 ratio with water for the final ashy standard.

### Statistical analysis

All statistical analysis was performed using R, version 4.2.1 “Funny-Looking Kid” (R Core Team, 2022) at a significance level of α = 0.05.

#### Descriptive data analysis

DA data was exported from Redjade, converting the position on the 15 cm line scale into scores from 0 to 100 for each attribute’s intensity ratings. A three-factor MANOVA (judge, product, replicate) was first performed across all attributes. Following, a three-factor ANOVA with two-way interactions between the panelists, replicates and products was performed to determine which attributes varied significantly among the wines. A pseudo-mixed ANOVA was then performed to determine which attributes were significant using the judge-by-product and replication-by-product interactions as the denominator when performing the test for significant product effects^30^. Fisher’s Least Significant Difference (LSD) test was used to compare attribute means for the significant attributes, using the “Agricolae” package.

Principal Component Analysis (PCA) biplots with 95% confidence ellipses was performed using the “SensomineR” package. Bootstrapping across the judges was used to display confidence ellipses around the products for a graphical representation of significant differences among wines^31^. Data was scaled to unit variance.

Multiple-factor analysis (MFA) was used to relate and compare different data sets; descriptive analysis, chemical composition, total volatile phenols, and individual bound phenolic glycosides to determine how similar wines were to each other across the different product spaces. Data was scaled to unit variance to account for scaling differences. The goodness of fit test of a MFA solution is a RV coefficient where RV values larger than 0.75 indicated strong relationships^32,33^. The “factomineR” package and “MFA” function were used to analyze the data and “ggplot2” was used for graphical representation.

## Results

### Chemical composition

The basic chemical composition of the wines is shown below in Table 2. There were significant differences across all variables measured. In particular, alcohol %, TA, and RS are deemed to have sensory implications^34,35^. The difference in the alcohol of the wines was driven primarily by the brix of the grapes that were harvested (Supplemental Table S4).

**Table 2:** Basic chemical composition of the wines (*n* = 3, α ≤ 0.05).

Wine	% Alcohol (%v/v)	pH	TA (g/L)	Residual Sugar (g/L)	Malic Acid (mg/L)	Acetic Acid ( g/L)
DA 1						
20CS_A_ST	16.11 ± 0.03a	3.70 ± 0.00d	5.56 ± 0.06c	0.83 ± 0.01a	142.67 ± 24.01ab	0.43 ± 0.06ab
20CS_B_ST	13.32 ± 0.03f	3.99 ± 0.02a	4.45 ± 0.02f	0.22 ± 0.02e	28.67 ± 49.65cd	0.36 ± 0.04cde
20CS_C_ST	13.71 ± 0.03e	3.47 ± 0.01e	5.49 ± 0.11c	0.29 ± 0.02d	40.00 ± 36.06cd	0.24 ± 0.04f
20CS_D_ST	11.80 ± 0.07 g	3.92 ± 0.01ab	5.24 ± 0.00d	0.15 ± 0.01g	4.00 ± 6.93d	0.38 ± 0.04bcd
20CS_E_ST	14.13 ± 0.03d	3.93 ± 0.01ab	5.18 ± 0.05d	0.25 ± 0.01e	4.67 ± 8.08d	0.42 ± 0.06bcd
20CS_F_ST	15.23 ± 0.01b	3.88 ± 0.01bc	4.91 ± 0.02e	0.35 ± 0.00b	77.33 ± 37.69c	0.46 ± 0.05a
20CS_G_ST	14.63 ± 0.53c	3.76 ± 0.19d	5.49 ± 0.34c	0.32 ± 0.01cd	29.00 ± 25.71cd	0.35 ± 0.06de
20CS_H_ST	15.25 ± 0.02b	3.95 ± 0.06ab	5.11 ± 0.04d	0.34 ± 0.01bc	24.50 ± 45.47d	0.38 ± 0.04bcd
21CS_I_NST	14.68 ± 0.02c	3.79 ± 0.01cd	6.01 ± 0.06b	0.10 ± 0.05h	46.67 ± 49.74cd	0.30 ± 0.01ef
21CS_J_NST	15.31 ± 0.01b	3.49 ± 0.02e	6.71 ± 0.07a	0.17 ± 0.01fg	173.67 ± 40.20a	0.33 ± 0.01de
21CS_K_ST	15.44 ± 0.02b	3.46 ± 0.01e	6.82 ± 0.08a	0.18 ± 0.01f	85.00 ± 28.69bc	0.34 ± 0.03de
DA 2						
20CF_A_ST	14.52 ± 0.53cd	3.83 ± 0.01ab	5.06 ± 0.08f	0.52 ± 0ef	6.00 ± 10.39c	0.39 ± 0.02cdef
20CF_B_ST	14.54 ± 1.06cd	3.96 ± 0.01a	4.81 ± 0.05f	0.50 ± 0.01f	27.67 ± 30.89bc	0.37 ± 0.13def
20CF_C_ST	15.28 ± 0.06b	3.74 ± 0.03bc	5.08 ± 0.05f	0.60 ± 0.06cde	62.67 ± 94.2abc	0.56 ± 0.03b
20MA_D_ST	15.53 ± 0.02b	3.63 ± 0.01 cd	6.62 ± 0.04bc	0.62 ± 0.00 cd	0.00 ± 0.00c	0.34 ± 0.02ef
20ME_E_ST	16.21 ± 0.17a	3.52 ± 0.01def	5.75 ± 0.07de	1.50 ± 0.00a	151.67 ± 13.87a	0.49 ± 0.12bc
20ME_F_ST	14.30 ± 0.03de	3.42 ± 0.02ef	6.09 ± 0.06cd	0.38 ± 0.02g	83 ± 83.83abc	0.24 ± 0.05g
20PV_G_ST	15.48 ± 0.26b	3.98 ± 0.03a	5.21 ± 0.09ef	0.66 ± 0.00c	0.00 ± 0.00c	0.47 ± 0.05bcd
20PV_H_ST	15.11 ± 0.01bc	3.97 ± 0.02a	5.11 ± 0.04ef	0.53 ± 0.00def	0.00 ± 0.00c	0.41 ± 0.03cdef
20SY_I_ST	14.90 ± 0.24bcd	3.60 ± 0.31cde	6.79 ± 1.34b	0.88 ± 0.19b	146 ± 141.71a	0.43 ± 0.01cde
20ZN_J_ST	15.13 ± 0.03bc	3.46 ± 0.13def	6.62 ± 0.05bc	0.67 ± 0.02c	89.33 ± 36.56abc	0.68 ± 0.11a
21MA_K_NST	15.26 ± 0.07b	3.34 ± 0.01f	7.84 ± 0.05a	0.39 ± 0.01g	0.00 ± 0.00c	0.33 ± 0.02fg
21SY_L_NST	13.74 ± 0.34e	3.51 ± 0.22def	6.53 ± 0.02bc	0.10 ± 0.02h	115 ± 101.68ab	0.40 ± 0.08cdef

Fisher’s LSD for means comparison of all samples across each chemical parameter. Letters that are different indicate a significant difference, *p* ≤ 0.05 in each DA panel.

### Free and total volatile phenols, and individual bound glycosides

The free VP, total VP, and individual bound glycoside compositions of the wines are shown in Tables 3 and 4, and 5, respectively. Across each DA panel set of wines, there were significant differences in the levels of free VPs, total VPs, and individual bound concentrations. Wines that were not smoke-tainted, 21CS_I_NST, 21CS_J_NST, 21MA_K_NST, and 21SY_L_NST had low levels of free VPs, total VPs, and individual bound glycoside concentrations in comparison to their smoke-tainted counterparts. In particular, 21CS_I_NST was significantly different from its smoked counterpart 20CS_E_ST, and 21CS_J_NST was significantly different from its smoked counterpart, 21CS_K_ST (Tables 3 and 4, and 5). The non-smoke impacted wines, in DA1, 21CS_I_NST and 21CS_J_NST, was not significantly different from each other. However, in DA 2, the 21MA_K_NST and 21SY_L_NST wines were significantly different from each other for most of the VP compounds (Tables 3 and 4, and 5) with high levels of VPs in the Syrah wine compared to the Malbec wine.

**Table 3:** Free volatile phenol concentrations of the wines.

Wine	Free_guaiacol	Free_4-methylguaiacol	Free_*o*-cresol	Free_phenol	Free_4-ethylguaiacol	Free_*p*-cresol	Free_*m*-cresol	Free_2,3-dimethoxyphenol	Free_4-ethylphenol	Free_syringol	Free_4-methylsyringol
DA 1											
20CS_A_ST	7.71 ± 0.33d	1.06 ± 0.06e	5.09 ± 0.19e	16.23 ± 0.53e	0.18 ± 0.00f	3.26 ± 0.19c	4.68 ± 0.21f	1.93 ± 0.08d	0.72 ± 0.03e	29.41 ± 0.46d	0.80 ± 0.07e
20CS_B_ST	7.42 ± 0.01d	1.96 ± 0.01d	5.08 ± 0.00e	23.31 ± 0.06b	0.61 ± 0.00c	4.03 ± 0.02b	5.35 ± 0.01e	7.77 ± 0.02b	1.10 ± 0.00c	42.05 ± 0.88c	3.93 ± 0.01c
20CS_C_ST	9.33 ± 0.04c	2.44 ± 0.02c	5.74 ± 0.02d	15.97 ± 0.05e	0.76 ± 0.00b	3.18 ± 0.01c	6.34 ± 0.05c	5.56 ± 0.02c	1.11 ± 0.01c	45.13 ± 0.49b	6.21 ± 0.03b
20CS_D_ST	1.92 ± 0.03f	0.34 ± 0.00f	1.56 ± 0.02f	3.98 ± 0.03g	0.08 ± 0.00g	1.01 ± 0.01f	1.18 ± 0.01 g	0.45 ± 0.00e	0.11 ± 0.01 g	15.61 ± 1.27f	0.27 ± 0.01ef
20CS_E_ST	33.20 ± 0.10b	5.59 ± 0.09b	20.93 ± 0.19b	72.42 ± 0.19a	0.44 ± 0.00d	16.7 ± 0.11a	22.17 ± 0.17b	19.05 ± 0.24a	2.92 ± 0.03a	18.85 ± 0.05e	1.80 ± 0.02d
20CS_F_ST	2.37 ± 0.05ef	0.33 ± 0.01f	1.31 ± 0.04f	3.12 ± 0.12h	0.12 ± 0.02 g	0.94 ± 0.11fg	0.95 ± 0.03gh	0.00 ± 0.00f	0.12 ± 0.02 fg	18.53 ± 1.12e	0.20 ± 0.03f
20CS_G_ST	9.77 ± 0.19c	1.85 ± 0.03d	6.41 ± 0.17c	18.60 ± 0.25d	0.32 ± 0.02e	2.84 ± 0.04d	5.78 ± 0.11d	5.58 ± 0.13c	0.68 ± 0.00e	12.51 ± 0.17g	1.52 ± 0.18d
20CS_H_ST	2.49 ± 0.03e	0.30 ± 0.02fg	1.35 ± 0.04f	8.05 ± 0.18f	0.16 ± 0.00f	2.25 ± 0.16e	0.80 ± 0.04 h	0.12 ± 0.01f	0.78 ± 0.04d	16.04 ± 0.17f	0.28 ± 0.00ef
21CS_I_NST	0.78 ± 0.02g	0.11 ± 0.00gh	0.56 ± 0.02g	2.92 ± 0.23h	0.02 ± 0.00h	0.79 ± 0.01g	0.43 ± 0.02i	0.00 ± 0.00f	0.16 ± 0.01f	11.35 ± 0.40g	0.00 ± 0.00f
21CS_J_NST	0.79 ± 0.01g	0.10 ± 0.00h	0.62 ± 0.02g	1.65 ± 0.15i	0.00 ± 0.00h	0.40 ± 0.01h	0.46 ± 0.03i	0.00 ± 0.00f	0.16 ± 0.01f	8.01 ± 0.20h	0.00 ± 0.00f
21CS_K_ST	81.45 ± 0.73a	31.47 ± 0.28a	22.31 ± 0.29a	20.52 ± 0.10c	5.49 ± 0.05a	0.37 ± 0.01h	34.76 ± 0.40a	0.00 ± 0.00f	2.65 ± 0.04b	138.9 ± 1.34a	77.57 ± 0.91a
DA 2											
20CF_A_ST	7.19 ± 0.35h	1.75 ± 0.13g	3.46 ± 0.14f	9.24 ± 0.31 g	0.09 ± 0.00g	4.34 ± 0.28c	3.78 ± 0.24d	0.02 ± 0.00f	0.86 ± 0.08e	38.63 ± 3.22d	0.42 ± 0.00e
20CF_B_ST	14.02 ± 0.63e	5.15 ± 0.24c	6.82 ± 0.30c	26.70 ± 1.8b	1.05 ± 0.03b	10.15 ± 0.97a	11.79 ± 0.84a	0.16 ± 0.00e	2.18 ± 0.16a	55.10 ± 6.79c	3.71 ± 0.20a
20CF_C_ST	2.34 ± 0.05i	0.32 ± 0.00hi	1.30 ± 0.04g	3.04 ± 0.04hi	0.10 ± 0.00 g	0.87 ± 0.00fg	0.94 ± 0.03e	0.00 ± 0.00f	0.10 ± 0.00 h	19.22 ± 0.69gh	0.04 ± 0.04fg
20MA_D_ST	19.97 ± 0.68d	5.74 ± 0.11b	9.24 ± 0.29a	25.17 ± 0.52c	0.43 ± 0.01c	6.5 ± 0.22b	9.35 ± 0.20b	0.4 ± 0.01c	1.55 ± 0.04c	21.85 ± 0.78fg	0.92 ± 0.04c
20ME_E_ST	1.61 ± 0.12j	0.14 ± 0.01i	0.53 ± 0.01i	1.90 ± 0.09i	0.10 ± 0.02g	0.51 ± 0.05 g	0.69 ± 0.02e	0.00 ± 0.00f	0.12 ± 0.00 h	27.09 ± 1.72ef	0.13 ± 0.00f
20ME_F_ST	10.20 ± 0.13g	2.90 ± 0.02f	4.14 ± 0.05e	11.03 ± 0.14f	0.27 ± 0.01d	2.6 ± 0.03e	4.93 ± 0.03c	0.32 ± 0.01d	0.57 ± 0.01 g	7.62 ± 0.25i	0.70 ± 0.02d
20PV_G_ST	25.31 ± 0.50c	4.13 ± 0.07e	5.79 ± 0.32d	12.88 ± 0.58e	0.25 ± 0.01d	3.47 ± 0.33d	5.34 ± 0.24c	2.09 ± 0.11a	0.70 ± 0.05f	108.26 ± 2.62a	0.72 ± 0.02d
20PV_H_ST	47.27 ± 0.58a	14.06 ± 0.18a	9.23 ± 0.07a	21.71 ± 0.05d	1.72 ± 0.02a	4.76 ± 0.05c	11.26 ± 0.03a	1.91 ± 0.03b	1.70 ± 0.02b	81.84 ± 7.80b	2.98 ± 0.02b
20SY_I_ST	34.56 ± 0.95b	4.63 ± 0.07d	8.82 ± 0.25b	33.62 ± 0.71a	0.21 ± 0.00e	5.93 ± 0.19b	9.53 ± 0.20b	0.41 ± 0.02c	1.27 ± 0.03d	29.29 ± 0.39e	0.88 ± 0.03c
20ZN_J_ST	1.91 ± 0.08ij	0.34 ± 0.01h	0.28 ± 0.01i	3.88 ± 0.04 h	0.17 ± 0.01f	1.18 ± 0.08f	0.81 ± 0.04e	0.00 ± 0.00f	0.63 ± 0.03 fg	7.42 ± 0.20i	0.20 ± 0.00f
21MA_K_NST	1.38 ± 0.02j	0.19 ± 0.00hi	0.52 ± 0.01i	2.49 ± 0.35i	0.03 ± 0.00h	0.57 ± 0.03fg	0.48 ± 0.01e	0.00 ± 0.00f	0.22 ± 0.00h	11.74 ± 0.28hi	0.00 ± 0.00g
21SY_L_NST	12.23 ± 0.07f	0.15 ± 0.00i	0.91 ± 0.02 h	3.11 ± 0.13hi	0.00 ± 0.00h	0.84 ± 0.01fg	0.39 ± 0.01e	0.00 ± 0.00f	0.22 ± 0.01h	9.01 ± 0.03i	0.00 ± 0.00g

All concentrations are in µg/L (*n* = 3).

Fisher’s LSD was used to determine differences between each column for each DA across wines.

**Table 4:** Acid-labile (total) volatile phenol concentrations of wines as determined by acid hydrolysis.

Wine	Total_guaiacol	Total_4-methylguaiacol	Total_*o*-cresol	Total_phenol	Total_4-ethylguaiacol	Total_*p*-cresol	Total_*m*-cresol	Total_2,3-dimethoxyphenol	Total_4-ethylphenol	Total_syringol	Total_4-methylsyringol
DA 1											
20CS_A_ST	16.88 ± 0.98f	3.03 ± 0.19e	7.65 ± 0.38e	25.02 ± 1.42de	0.55 ± 0.04g	7.40 ± 0.38cd	9.14 ± 0.52e	2.80 ± 0.45e	4.94 ± 0.40e	73.3 ± 4.90e	17.99 ± 2.49f
20CS_B_ST	23.74 ± 0.69e	6.54 ± 0.34d	10.48 ± 0.55d	40.25 ± 2.15c	1.31 ± 0.07d	8.62 ± 0.40c	13.47 ± 0.70d	30.79 ± 1.30b	10.5 ± 1.26d	104.96 ± 7.87d	25.68 ± 0.17e
20CS_C_ST	40.97 ± 0.85c	14.34 ± 0.83c	18.63 ± 1.12c	51.72 ± 3.64b	2.09 ± 0.09b	11.9 ± 0.59b	26.65 ± 1.31c	25.11 ± 0.25c	17.5 ± 2.16b	158.92 ± 5.50c	57.11 ± 0.31d
20CS_D_ST	9.23 ± 0.29g	2.26 ± 0.03ef	4.30 ± 0.15f	15.86 ± 0.88f	0.79 ± 0.03f	5.53 ± 0.03e	4.59 ± 0.08f	1.43 ± 0.06f	8.56 ± 0.23d	45.03 ± 1.47f	3.44 ± 0.10g
20CS_E_ST	84.38 ± 0.85b	19.66 ± 0.82b	33.42 ± 1.23b	112.16 ± 3.32a	1.30 ± 0.04d	33.45 ± 1.43a	47.76 ± 2.07b	31.88 ± 0.44a	14.14 ± 1.39c	415.38 ± 2.77a	211.29 ± 0.45a
20CS_F_ST	3.85 ± 0.13i	0.70 ± 0.03f	1.72 ± 0.12g	3.16 ± 0.04g	0.25 ± 0.00 h	1.51 ± 0.03f	1.65 ± 0.12g	0.00 ± 0.00g	1.42 ± 0.05f	39.65 ± 0.08f	1.17 ± 0.06h
20CS_G_ST	31.75 ± 2.25d	7.97 ± 0.69d	9.30 ± 0.66de	28.01 ± 1.76de	0.95 ± 0.08e	8.38 ± 0.76cd	11.56 ± 0.97de	8.87 ± 0.72d	3.80 ± 0.56ef	109.66 ± 0.44d	72.73 ± 0.17c
20CS_H_ST	6.02 ± 0.61h	1.45 ± 0.20ef	3.23 ± 0.44fg	23.77 ± 1.41e	1.79 ± 0.12c	7.31 ± 0.09d	3.81 ± 0.18fg	0.43 ± 0.02 fg	19.56 ± 1.3b	21.67 ± 0.54g	1.44 ± 0.10h
21CS_I_NST	6.60 ± 0.60h	0.94 ± 0.02f	2.56 ± 0.24g	25.45 ± 0.14de	0.58 ± 0.01g	4.45 ± 0.41e	3.52 ± 0.26fg	0.00 ± 0.00g	17.54 ± 0.20b	22.76 ± 0.84g	0.32 ± 0.00h
21CS_J_NST	6.98 ± 0.37h	1.07 ± 0.11f	2.98 ± 0.18fg	29.34 ± 0.11d	0.92 ± 0.09ef	4.49 ± 0.32e	4.20 ± 0.20fg	0.00 ± 0.00g	24.38 ± 3.53a	20.62 ± 0.19g	0.53 ± 0.05h
21CS_K_ST	96.8 ± 1.37a	53.26 ± 2.15a	39.28 ± 1.72a	50.03 ± 3.05b	6.82 ± 0.14a	5.00 ± 0.59e	65.36 ± 3.56a	0.00 ± 0.00g	18.19 ± 1.51b	181.27 ± 1.8b	88.64 ± 0.48b
DA 2											
20CF_A_ST	27.54 ± 0.32g	9.56 ± 0.50e	10.25 ± 0.46d	36.56 ± 2.81de	1.70 ± 0.12d	12.40 ± 0.59c	12.64 ± 0.43d	0.48 ± 0.00g	32.76 ± 4.05a	128.35 ± 5.35d	25.39 ± 0.04e
20CF_B_ST	54.39 ± 1.15f	23.85 ± 1.90d	16.06 ± 1.29b	45.12 ± 1.82b	2.60 ± 0.08b	19.05 ± 0.91b	26.78 ± 0.96b	1.12 ± 0.11f	12.36 ± 0.84b	196.88 ± 11.43b	77.05 ± 1.16b
20CF_C_ST	4.15 ± 0.43i	0.77 ± 0.10f	1.72 ± 0.11h	3.16 ± 0.04g	0.25 ± 0.00g	1.51 ± 0.03h	1.63 ± 0.10g	0.00 ± 0.00h	1.45 ± 0.10e	69.32 ± 4.53e	1.23 ± 0.14h
20MA_D_ST	88.77 ± 0.61d	37.92 ± 0.18b	21.45 ± 0.36a	63.32 ± 0.62a	2.17 ± 0.02c	25.42 ± 0.17a	29.30 ± 0.40a	1.83 ± 0.03e	12.43 ± 0.11b	153.57 ± 1.70c	54.85 ± 0.63d
20ME_E_ST	6.55 ± 0.14hi	1.40 ± 0.03f	1.63 ± 0.06h	4.21 ± 0.15g	0.33 ± 0.01g	2.90 ± 0.07gh	2.02 ± 0.07g	0.00 ± 0.00h	1.62 ± 0.17e	49.14 ± 70ef	1.56 ± 0.11h
20ME_F_ST	96.44 ± 5.87c	37.98 ± 3.63b	13.64 ± 1.67c	39.93 ± 4.55cd	2.10 ± 0.11c	17.88 ± 2.23b	22.67 ± 2.41c	2.31 ± 0.13c	4.84 ± 0.32de	180.18 ± 3.17b	134.3 ± 2.30a
20PV_G_ST	52.00 ± 0.89f	11.00 ± 0.89e	8.57 ± 0.63e	20.35 ± 1.41f	0.74 ± 0.09f	7.03 ± 1.00e	10.54 ± 0.83e	4.68 ± 0.13b	5.51 ± 0.38d	192.08 ± 11.24b	16.66 ± 2.21f
20PV_H_ST	115.79 ± 2.09b	44.57 ± 2.05a	15.04 ± 0.68bc	34.43 ± 1.88e	3.74 ± 0.07a	10.26 ± 0.70d	21.94 ± 0.92c	6.29 ± 0.15a	8.41 ± 1.06 cd	222.74 ± 38.71a	75.56 ± 0.62b
20SY_I_ST	171.54 ± 5.65a	34.7 ± 1.94c	21.41 ± 0.99a	62.59 ± 1.94a	1.04 ± 0.03e	25.28 ± 1.61a	28.21 ± 1.40ab	2.00 ± 0.04d	11.28 ± 0.88bc	148.29 ± 0.13cd	60.50 ± 0.10c
20ZN_J_ST	7.54 ± 0.23hi	2.05 ± 0.05f	4.36 ± 0.26g	16.05 ± 0.57f	1.85 ± 0.24d	7.21 ± 0.27e	5.99 ± 0.15f	0.00 ± 0.00h	10.62 ± 1.44bc	12.44 ± 0.27 g	3.92 ± 0.24g
21MA_K_NST	10.79 ± 0.47h	2.79 ± 0.14f	3.50 ± 0.21g	41.72 ± 4.61bc	0.99 ± 0.07e	6.76 ± 0.45ef	6.45 ± 0.30f	0.00 ± 0.00h	31.07 ± 3.47a	26.52 ± 0.32fg	0.62 ± 0.01h
21SY_L_NST	82.28 ± 1.59e	2.49 ± 0.12f	6.87 ± 0.41f	32.03 ± 2.98e	0.56 ± 0.05f	4.82 ± 0.37fg	4.91 ± 0.12f	0.00 ± 0.00h	29.08 ± 3.01a	20.35 ± 0.11g	0.70 ± 0.02h

All concentrations are in µg/L.

Fisher’s LSD was used to determine differences between each column for each DA across wines.

**Table 5:** Individual bound glycoside concentrations of wines.

Wine	Guaiacol gentiobioside	Syringol gentiobioside	Guaiacol glucoside	Phenol rutinoside	Guaiacol rutinoside	4-Methylsyringol gentiobioside	Cresol rutinoside	4-Methylguaiacol rutinoside
DA 1								
20CS_A_ST	0.55 ± 0.02e	35.58 ± 0.24d	6.16 ± 0.17f	15.62 ± 0.4d	5.58 ± 0.20d	4.70 ± 0.05d	7.75 ± 0.01e	3.10 ± 0.04e
20CS_B_ST	0.84 ± 0.04d	26.21 ± 0.34e	17.21 ± 0.26e	15.90 ± 0.30d	5.67 ± 0.25d	2.82 ± 0.13e	9.5 ± 0.11d	3.69 ± 0.04d
20CS_C_ST	3.62 ± 0.07b	78.36 ± 0.25c	38.22 ± 0.41a	27.02 ± 0.32c	14.78 ± 0.27c	11.85 ± 0.12c	15.01 ± 0.45c	10.22 ± 0.13c
20CS_D_ST	0.06 ± 0.00f	7.42 ± 0.10f	1.57 ± 0.01 g	4.68 ± 0.12f	1.42 ± 0.03e	0.63 ± 0.02f	3.80 ± 0.12 g	1.05 ± 0.01 g
20CS_E_ST	3.03 ± 0.07c	448.29 ± 2.77a	33.68 ± 0.16b	53.51 ± 0.37a	32.84 ± 0.13a	48.61 ± 0.46a	42.49 ± 0.39a	20.88 ± 0.24a
20CS_F_ST	0.04 ± 0.00f	2.41 ± 0.06 h	0.74 ± 0.01hi	3.24 ± 0.11 g	0.68 ± 0.03f	0.10 ± 0.00 g	1.43 ± 0.03 h	0.13 ± 0.00i
20CS_G_ST	3.92 ± 0.11a	105.06 ± 1.09b	23.92 ± 0.19c	39.86 ± 0.30b	25.30 ± 0.20b	18.65 ± 0.6b	27.35 ± 0.55b	14.00 ± 0.19b
20CS_H_ST	0.09 ± 0.00f	4.49 ± 0.08 g	0.99 ± 0.06 h	8.68 ± 0.33e	1.46 ± 0.10e	0.29 ± 0.01 fg	4.72 ± 0.05f	0.47 ± 0.03 h
21CS_I_NST	0.02 ± 0.00f	1.34 ± 0.07 h	0.55 ± 0.00i	1.28 ± 0.00i	0.32 ± 0.01 g	0.11 ± 0.01 g	1.24 ± 0.07 h	0.50 ± 0.03 h
21CS_J_NST	0.06 ± 0.01f	2.21 ± 0.04 h	0.40 ± 0.02i	2.25 ± 0.06 h	0.53 ± 0.03 fg	0.16 ± 0.01 g	1.12 ± 0.03 h	0.31 ± 0.01hi
21CS_K_ST	0.59 ± 0.01e	7.71 ± 0.08f	21.9 ± 0.30d	2.37 ± 0.06 h	0.71 ± 0.01f	2.67 ± 0.04e	1.48 ± 0.02 h	1.30 ± 0.02f
DA 2								
20CF_A_ST	2.60 ± 0.23f	36.53 ± 1.67e	11.47 ± 0.27 h	3.24 ± 0.06 h	1.39 ± 0.09hi	5.01 ± 0.34f	4.36 ± 0.18 g	2.17 ± 0.04 g
20CF_B_ST	6.68 ± 0.35c	84.95 ± 3.84d	42.96 ± 0.07e	5.25 ± 0.34 g	2.01 ± 0.01 g	14.08 ± 1.00d	11.08 ± 0.25e	3.98 ± 0.06f
20CF_C_ST	0.45 ± 0.02 h	4.82 ± 0.02 g	2.04 ± 0.05j	1.54 ± 0.11i	0.42 ± 0.02k	0.19 ± 0.00i	1.78 ± 0.13i	0.50 ± 0.03j
20MA_D_ST	3.90 ± 0.03e	110.03 ± 1.49b	48.34 ± 0.14d	41.25 ± 0.57c	20.83 ± 0.28c	17.74 ± 0.17c	29.97 ± 0.47c	18.61 ± 0.22c
20ME_E_ST	0.42 ± 0.04 h	2.80 ± 0.07 g	2.45 ± 0.06j	3.38 ± 0.15 h	0.95 ± 0.05j	0.24 ± 0.02i	3.27 ± 0.23 h	1.23 ± 0.01i
20ME_F_ST	50.0 ± 0.02a	224.12 ± 2.35a	66.04 ± 1.16b	42.81 ± 0.59b	21.70 ± 0.48b	45.1 ± 0.94a	52.45 ± 0.54b	29.22 ± 0.15b
20PV_G_ST	1.06 ± 0.07 g	37.90 ± 1.53e	18.42 ± 0.32 g	9.81 ± 0.15f	8.70 ± 0.21e	2.81 ± 0.12 g	8.26 ± 0.29f	8.14 ± 0.02e
20PV_H_ST	3.65 ± 0.1e	82.44 ± 0.88d	52.60 ± 0.6c	12.31 ± 0.24e	13.67 ± 0.28d	12.84 ± 0.50e	14.58 ± 0.30d	15.69 ± 0.12d
20SY_I_ST	27.21 ± 0.34b	104.58 ± 0.77c	121.40 ± 0.59a	65.24 ± 1.11a	40.27 ± 0.06a	22.98 ± 0.20b	59.67 ± 0.89a	34.89 ± 0.24a
20ZN_J_ST	1.28 ± 0.03 g	8.27 ± 0.17f	3.85 ± 0.04i	17.32 ± 0.83d	1.75 ± 0.05gh	1.44 ± 0.06 h	7.86 ± 0.20f	1.15 ± 0.02i
21MA_K_NST	0.19 ± 0.01 h	2.96 ± 0.04 g	1.07 ± 0.03k	1.85 ± 0.10i	1.28 ± 0.02ij	0.19 ± 0.01i	2.30 ± 0.02i	2.06 ± 0.06gh
21SY_L_NST	4.94 ± 0.04d	2.69 ± 0.02 g	31.48 ± 0.16f	1.27 ± 0.13i	7.35 ± 0.15f	0.28 ± 0.00i	2.34 ± 0.09i	1.89 ± 0.04 h

All concentrations are in µg/L.

Fisher’s LSD was used to determine differences between each column for each DA across wines.

### Sensory profile

**Fig. 1:**
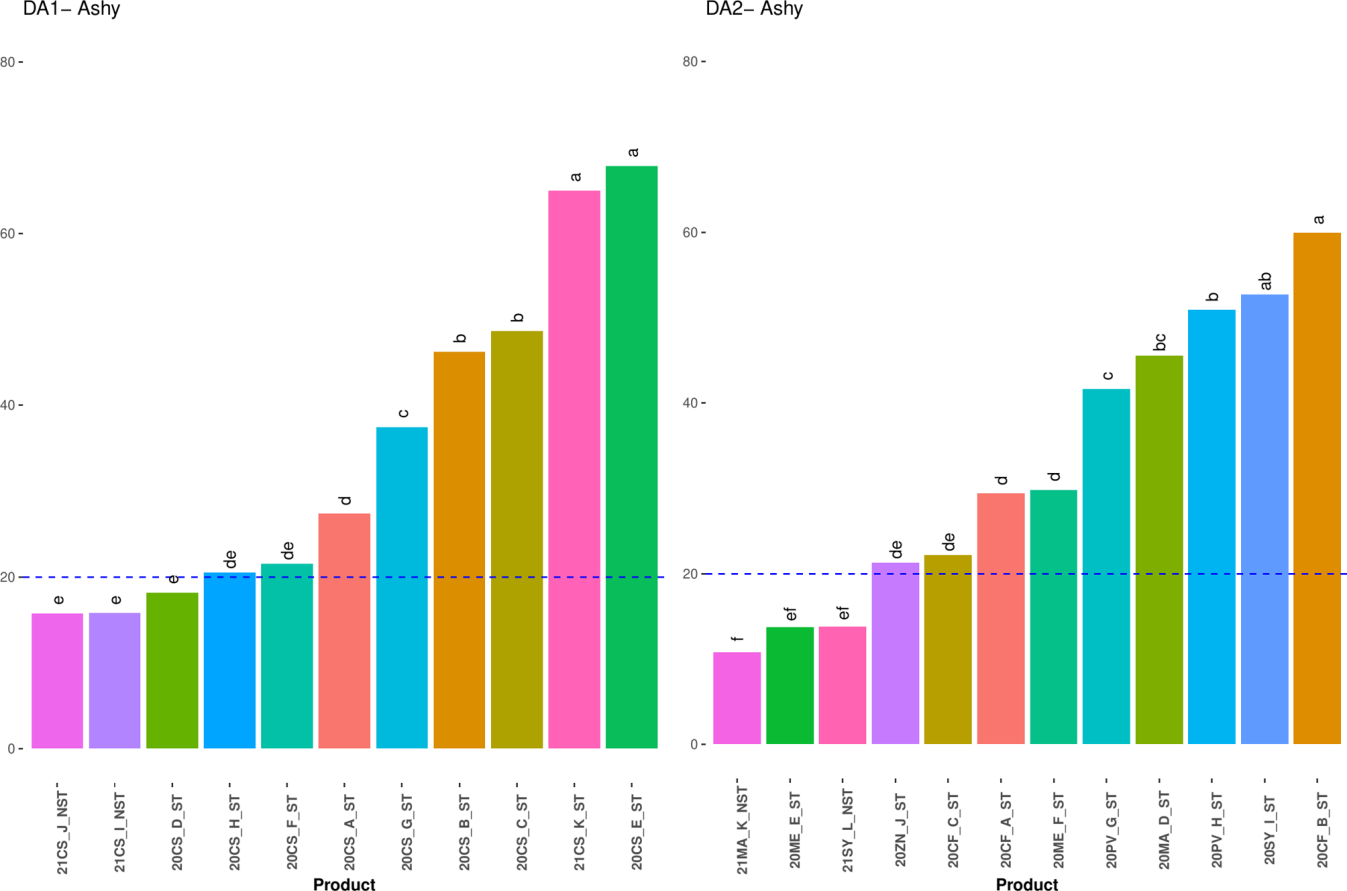
Mean attribute intensities of “Ashy” attribute in DA1 and DA2 wines, with Fisher’s LSD (α = 0.05).

Trained panelists evaluated across two panels (DA1, *n* = 14 judges, DA2, *n* = 13 judges) fifteen aroma, six taste/mouthfeel and one ashy retronasal attribute for a total of 22 attributes. In DA1, the wines differed significantly across twelve attributes and in DA 2 across nine attributes, as analyzed through a pseudo-mixed model ANOVA. The attribute means and Fisher’s Least Significant Difference (LSD) are shown in Tables 6 and 7, respectively. The “ashy” attribute can be defined as an indicator of the amount of smoke taint as it is the single characteristic unique to smoke-tainted wines^5,14^. Here, the NST wines were rated as having a low level of ashy (Fig. 1). Based on the NST data, a level up to 20 out of a 100 is generally seen as no smoke taint. The low ashy rating could have been due to cross-over effects between the samples. This was seen before in studies by Oberholster et al. and Fryer et al.^18,29^.

**Table 6:** DA 1 sensory attribute overall means with Fisher’s LSD.

Wine	Red fruit	Dark fruit	Alcohol hotness	Medicinal/Brett	Liquid smoke	Sweet BBQ	Tar	Viscosity	Drying	Sweet	Sour	Ashy
Significant attributes												
20CS_A_ST	14.64 bc	18.02 abc	56.93 a	17.43 bcd	11.62 cd	11.55 bcd	8.48 d	28.19 ab	48.05 ef	24.86 a	44.976 b	27.38 de
20CS_B_ST	14.93 bc	19.74 abc	47.52 bcde	21.62 abc	16.76 bc	16.74 b	10.26 cd	27.24 ab	50.86 de	16.81 bc	36.93 c	46.24 bc
20CS_C_ST	16.79 abc	16.10 bcd	43.29 cde	25.64 ab	20.31 b	15.14 bc	15.91 bc	21.55 ab	64.17 ab	12.88 c	43.67 bc	48.64 b
20CS_D_ST	23.72 a	18.64 abc	38.76 e	12.02 d	3.57 e	8.91 cd	4.31 d	19.79 b	66.48 a	10.95 c	38.86 bc	18.19 ef
20CS_E_ST	11.38 c	13.19 cd	41.14 de	27.74 a	33.31 a	28.24 a	17.33 ab	22.57 ab	59.41 abcd	12.36 c	40.57 bc	67.86 a
20CS_F_ST	25.07 a	24.12 a	53.60 ab	11.64 d	3.93 e	5.07 d	4.64 d	29.24 ab	51.41 cde	21.52 ab	42.91 bc	21.55 ef
20CS_G_ST	20.36 ab	21.05 ab	50.33 abc	13.38 cd	7.67 de	8.57 cd	7.79 d	23.38 ab	60.60 ab	16.36 bc	46.10 ab	37.45 cd
20CS_H_ST	22.39 ab	17.20 abcd	49.10 abcd	16.93 bcd	10.12 cde	7.49 d	6.83 d	30.33 a	41.20 f	24.21 ab	42.74 bc	20.50 ef
21CS_I_NST	22.45 ab	18.19 abc	49.26 abcd	14.69 cd	3.88 e	7.31 d	8.12 d	25.81 ab	60.41 abc	13.43 c	53.57 a	15.79 f
21CS_J_NST	24.38 a	19.31 abc	51.17 abc	15.64 cd	4.33 e	8.98 cd	8.07 d	26.60 ab	55.21 bcde	16.95 bc	52.95 a	15.76 f
21CS_K_ST	14.52 bc	9.43 d	43.43 cde	29.10 a	38.05 a	30.17 a	23.50 a	28.91 ab	50.48 de	16.81 bc	53.24 a	65.00 a

Wine	Cooked Fruit	Dried Fruit	Bell Pepper	Spice	Cigarette Smoke	Musty	Menthol	Solvent	Alcohol Hotness- Mouthfeel	Bitter
Non-significant attributes										
20CS_A_ST	15.33	17.60	5.26	10.29	6.88	17.50	18.88	33.60	56.43	45.31
20CS_B_ST	16.43	17.67	17.50	8.79	18.36	17.45	17.33	30.10	44.14	46.79
20CS_C_ST	16.10	17.40	12.12	7.57	16.12	21.26	16.26	27.90	44.88	43.64
20CS_D_ST	27.26	21.50	9.71	7.76	5.55	15.95	17.29	25.07	37.57	37.90
20CS_E_ST	11.79	10.57	5.95	5.76	24.57	27.64	15.69	24.00	49.45	46.55
20CS_F_ST	27.00	20.90	7.74	11.64	5.02	11.05	16.69	33.76	56.50	46.21
20CS_G_ST	21.76	21.31	6.69	8.88	8.36	17.79	18.93	32.24	52.83	45.19
20CS_H_ST	24.32	14.46	9.17	9.83	12.24	18.29	17.32	29.75	53.81	44.37
21CS_I_NST	26.05	19.93	8.36	10.50	4.71	17.05	16.71	36.74	54.12	40.17
21CS_J_NST	22.07	17.76	8.55	8.60	5.48	18.48	20.17	31.24	51.24	43.48
21CS_K_ST	8.83	13.33	4.14	6.88	25.48	20.07	14.45	27.55	52.83	43.50

Fisher’s LSD was used to determine differences between each column for the significant attributes.

**Table 7:** DA 2 sensory attribute overall means with Fisher’s LSD.

Wine	Red fruit	Bell pepper	Liquid smoke	Sweet BBQ	Solvent	Viscosity	Drying	Bitter	Ashy
Significant attributes									
20CF_A_ST	11.85 de	21.39 a	13.46 c	9.49 de	22.70 a	26.97 a	51.26 def	51.33 a	29.41 d
20CF_B_ST	11.51 de	9.62 b	17.51 bc	20.82 bc	21.41 a	27.95 a	46.28 f	52.56 a	59.92 a
20CF_C_ST	16.92 bcde	22.13 a	3.80 d	2.95 e	31.49 a	24.95 a	61.30 ab	50.82 a	22.21 de
20MA_D_ST	11.82 de	6.05 b	25.03 a	25.36 b	22.46 a	26.18 a	61.13 abc	50.90 a	45.56 bc
20ME_E_ST	21.41 abc	11.33 b	3.36 d	6.03 e	28.18 a	25.26 a	63.59 ab	44.87 a	13.72 ef
20ME_F_ST	17.44 bcde	5.64 b	4.23 d	7.87 e	29.44 a	21.13 a	68.26 a	48.13 a	29.82 d
20PV_G_ST	10.10 de	6.67 b	14.56 c	25.67 b	29.13 a	30.15 a	56.46 bcde	50.00 a	41.62 c
20PV_H_ST	13.97 cde	5.95 b	16.90 bc	16.62 cd	23.82 a	24.51 a	58.28 bcd	47.69 a	50.90 abc
20SY_I_ST	9.51 e	8.44 b	23.10 ab	44.80 a	22.10 a	25.85 a	52.82 cdef	43.97 a	52.74 ab
20ZN_J_ST	18.49 abcd	8.03 b	4.97 d	6.87 e	25.87 a	28.59 a	48.62 ef	44.31 a	21.28 def
21MA_K_NST	26.15 a	5.97 b	4.95 d	6.21 e	28.39 a	19.67 a	67.39 a	45.03 a	10.80 f
21SY_L_NST	23.03 ab	7.13 b	3.92 d	4.41 e	31.51 a	22.77 a	52.61 def	44.31 a	13.82 ef

Wine	Dark fruit	Cooked fruit	Dried fruit	Spice	Alcohol hotness	Cigarette smoke	Musty	Medicinal/Brett	Menthol	Tar	Alcohol hotness- mouthfeel	Sweet	Sour
Non-significant attributes													
20CF_A_ST	20.46	8.87	16.77	8.59	50.79	12.92	23.77	18.49	17.79	7.05	56.74	19.36	47.85
20CF_B_ST	19.26	13.28	20.74	8.41	50.64	17.79	21.08	21.05	14.18	8.05	55.77	19.82	40.85
20CF_C_ST	18.00	17.44	18	6.18	49.03	5.64	16.87	10.97	18.95	5.08	57.74	19.15	44.31
20MA_D_ST	15.72	15.18	17.13	10.13	50.64	13.33	17.85	15.69	14.82	9.72	56.38	16.97	53.00
20ME_E_ST	18.08	17.08	18.08	8.28	49.92	7.21	13.10	15.59	20.64	6.33	50.36	16.79	55.15
20ME_F_ST	22.36	23.72	17.56	9.79	54.72	2.74	12.44	9.62	18.97	6.08	62.69	21.05	53.03
20PV_G_ST	16.21	12.15	22.51	9.26	53.08	11.92	23.18	19.49	15.03	8.41	61.59	19.9	48.13
20PV_H_ST	15.18	13.26	19.46	7.26	49.36	15.44	23.13	21.36	19.46	6.77	54.62	19.41	45.41
20SY_I_ST	11.67	11.13	20.54	11.08	46.05	14.85	17.90	16.79	17.28	8.28	53.41	19.79	63.33
20ZN_J_ST	20.44	19.41	21.05	10.87	51.00	7.51	20.03	14.44	17.08	4.36	52.82	19.51	53.74
21MA_K_NST	18.54	23.67	18.28	10.97	51.31	5.26	13.49	10.13	17.87	2.21	54.87	14.97	63.21
21SY_L_NST	20.64	24.05	19.82	12.77	48.97	4.08	15.38	14.62	15.08	5.56	51.51	17.49	60.38

Fisher’s LSD was used to determine differences between each column for the significant attributes.

**Fig. 2:**
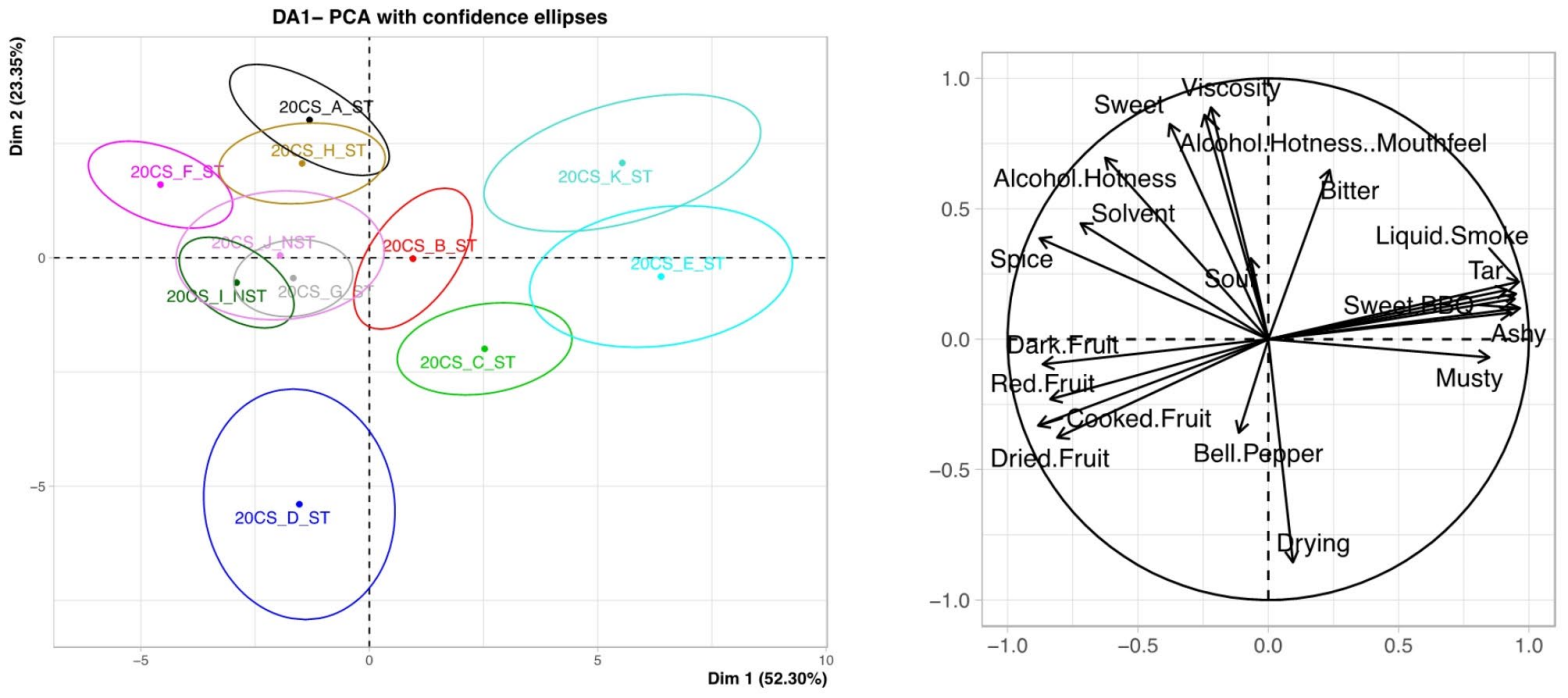
Score plot and loadings plot of DA1 with confidence ellipses around wines at 95% CI.

A PCA with confidence ellipses around the wine is shown in Fig. 2 for DA1. All attributes were considered. Overlapping circles mean that the wines are not significantly different from each other at a 95% confidence interval. Across PC1 (52.30%) and PC2 (23.35%) which explains 75.65% of the data, there is a clear separation of the wines across PC1. 20CS_F_ST, 21CS_I_NST, 21CS_J_NST and 20CS_G_ST are not significantly different from each other, these wines were “dark fruit”, “cooked fruit”, “red fruit”, and “dried fruit” driven. 20CS_A_ST and 20CS_H_ST are not significantly different from each other and were “sweet”, “viscosity”, “alcohol hotness” driven. 20CS_E_ST and 21CS_K_ST are not significantly different from each other, with these wines being, “musty”, “sweet BBQ”, “tar”, “ashy” and “liquid smoke” driven. 20CS_D_ST is significantly different from the rest of the wines with it being “drying” driven, confirmed by the mean values (Table 6).

**Fig. 3:**
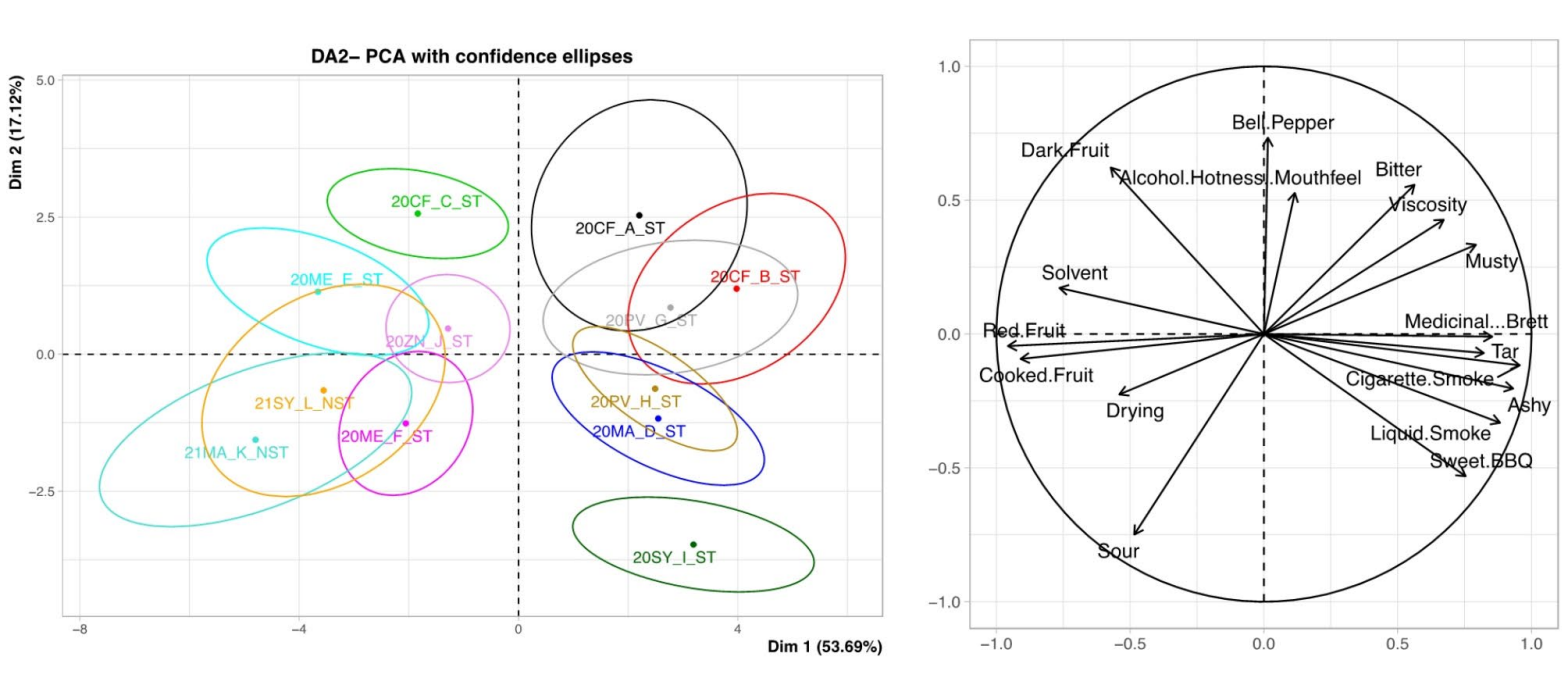
Score plot and loadings plot of DA2 with confidence ellipses around wines at 95% CI.

For DA2 in Fig. 3, across PC1 (53.69%) and PC2 (17.12%) which explains 70.81% of the data, there is a clear separation of the wines across PC1. 20ME_F_ST, 21MA_K_NST and 21SY_L_NST are not significantly different from each other, these wines were “cooked fruit”, “red fruit”, “drying” and “sour” driven. 21SY_L_NST, 20ZN_J_ST, and 20ME_E_ST are not significantly different from each other and were “dark fruit”, “solvent”, and “earthy” driven. 20CF_A_ST, 20CF_B_ST, 20MA_D_ST and 20PV_H_ST are not significantly different from each other, with these wines being, “musty”, “bitter”, “tar”, “cigarette smoke”, “ashy” and “liquid smoke” driven. 20SY_I_ST and 20CF_C_ST are significantly different from the rest of the wines with different smoke attributes and bell pepper attributes respectively. 20SY_I_ST had a high mean score for “sweet BBQ” at 44.80 while 20CF_C_ST had a mean score for “bell pepper” at 21.39. 20CF_C_ST differed from 20CF_A_ST and 20CF_B_ST as it had a higher “red fruit” score and lower smoke-related attribute mean scores (Table 7).

### Combining sensory and compositional data

**Fig. 4:**
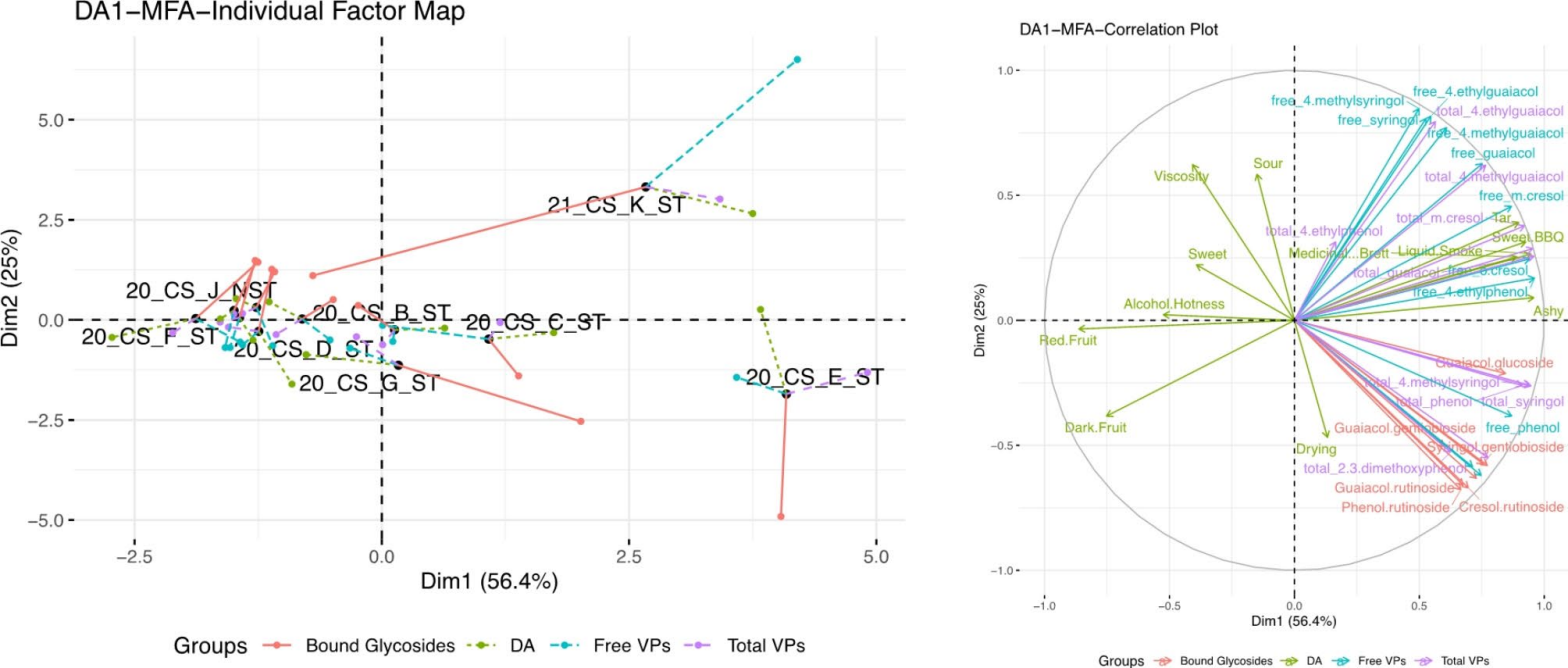
Multiple factor analysis of DA1 data, relating individual bound glycosides, significant attributes from the DA, free VPs, and total VPs. Individual factor map and loading plots are shown here.

Multiple factor analysis was used to relate sensory data to the chemical data of the wines. The free VPs, total VPs, individual bound glycosides, and significant attributes of the sensory data were analyzed using the MFA. Figure 4 shows the MFA of DA1. Wines were distinguished in the individual factor map similarly to the PCA in Fig. 2. The first two dimensions explain a total of 81.4% of the data with 56.4% across Dim1 and 25.0% across Dim2. In the loading plot, it is observed that the smoke-related DA attributes were driven by mainly free and total VPs. This was further confirmed by RV coefficients, where large RV coefficients indicate a good fit of the data. Between the DA and total VP, it has a RV coefficient of 0.857, DA and free VP at 0.758, and DA and bound glycosides at 0.404. However, the overall fit based on RV coefficients of the DA, total VPs, free VPs, and bound glycosides to the consensus positions were at 0.884, 0.972, 0.895, and 0.687. This indicates a good overall fit of the data. Certain VPs “free *o-*cresol”, “total guaiacol”, “free 4-ethylphenol”, “free *m*-cresol”, “total *o*-cresol”, and “total *m*-cresol” drive the smoke-related attributes, “tar”, “sweet BBQ”, “liquid smoke” and “ashy”. High smoke-impacted wines such as 21CS_K_ST and 20CS_E_ST were more smoke-driven sensorially by attributes (“tar”, “sweet BBQ”, and “ashy”), and chemically by free VPs, total VPs and bound glycosides. Contrary, low/no impact wines such as “20CS_F_ST” and “21CS_J_NST” were fruit-driven by the terms “red fruit” and “dark fruit” and was not driven by free VPs, total VPs and bound glycosides.

**Fig. 5:**
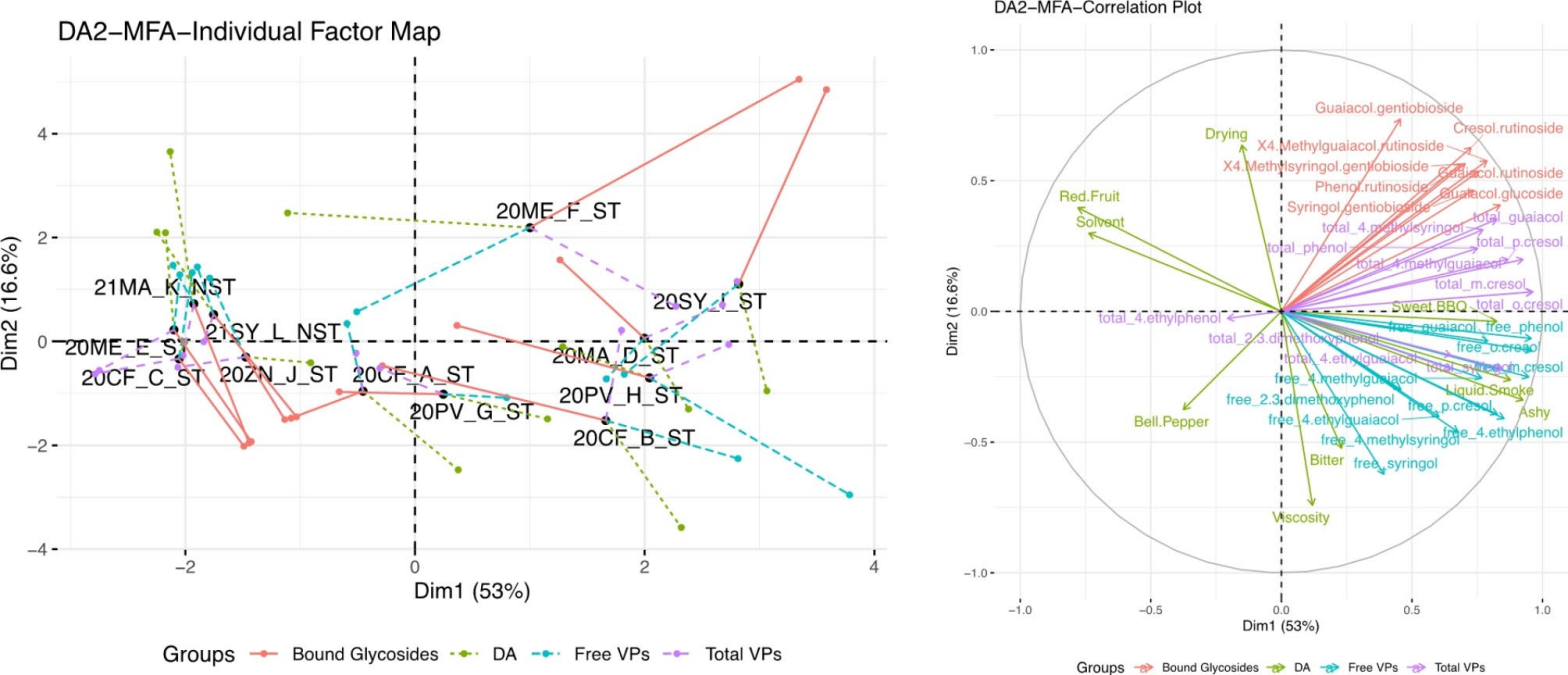
Multiple factor analysis of DA2 data, relating individual bound glycosides, significant attributes from the DA, free VPs, and total VPs. Individual factor map and loading plots are shown here.

Figure 5 shows the MFA of DA2. The first two dimensions explain a total of 69.6% of the overall data with 53.0% across Dim1 and 16.6% across Dim2. The overall spread of the wines in the individual factor map of the MFA is similar to the PCA biplot in Fig. 3. Here it was observed that the free VPs are the main drivers of the DA attributes. The RV coefficients for the DA and total VPs was 0.591, DA and free VPs was 0.674, and DA and bound glycosides was 0.313. Importantly, RV coefficients of the DA, total VPs, free VPs, and bound glycosides to the MFA consensus positions are at 0.805, 0.923, 0.829, and 0.681. When comparing the RV with the overall MFA plot, the total VPs are a better explanation for the DA attributes. This is seen when “total 2,3-dimethoxyphenol”, “total syringol”, “total *o*-cresol”, “total *p*-cresol”, “total *m*-cresol”, and “total ethylguaiacol” are key drivers of “liquid smoke”, “sweet BBQ”, and “ashy” attributes. Additionally, free VPs such as “free *o-*cresol”, “free *m-*cresol”, “free phenol”, and “free guaiacol” also contributed to the smoke DA attributes “liquid smoke”, “ashy”, and “sweet BBQ” which was seen in the relatively high RV coefficient values. Higher impacted wines, 20SY_I_ST, 20MA_D_ST, 20PV_H_ST and 20CF_B_ST were driven by more smoke-related DA attributes as well as the total and free VP amounts. Low/no impact wines such as 21MA_K_NST and 21SY_L_NST were “red fruit” and “dark fruit” driven overall.

## Discussion

There were significant differences in the basic wine chemical analysis, specifically the alcohol %, TA, and RS of the different wines which can have sensory implications^34,35^. However, “alcohol hotness”, “sweet”, and “sour” descriptor terms were significant only in DA1, determined by the pseudo-mixed ANOVA model (Fig. 4). When the DA1 data was normalized against the “alcohol hotness”, “sour” and “sweet” terms, it did not give a different result. Hence for the rest of the analysis, the effect of alcohol %, TA and RS was not considered, and the original data was used without normalization.

The determination of smoke impact could be quantified in the following ways, chemical analysis of smoke marker compounds, sensory analysis or a combination of both. Chemical analysis of smoke marker compounds in the free VP, total VP and the individual bound glycosides would give us an idea of the compositional impact on smoke. However, sensory analysis would need to be done on the wine to determine if there is indeed a smoke impact. In our experiment, there were significant chemical differences among the non-impacted and low-impacted, and highly impacted wines. The smoke impact can be determined using a trained descriptive analysis panel. The wines that were highly smoke impacted as determined by high values of VPs and the individual bound glycosides also were described by the sensory panel to be “ashy”, “smoky”, “sweet BBQ”. These wines lacked overall flavor intensity with a decrease in attributes such as “red fruit”, “dark fruit” and “jam fruit”.

In general, the impact of smoke on the wines were largely separated (> 50%) along PC1 for all the analyses presented. In the PCA plots shown in Figs. 2 and 3, wines that had higher levels of smoke marker compounds were grouped together and the non-tainted or those with low concentrations of smoke marker compounds were on the opposite ends of the PCA^8^. The wines that had higher smoke impact determined by higher concentrations of smoke marker compounds were described as more smoky and ashy. Contrary, wines that were not smoke-impacted or low impacted as determined by low concentrations of smoke marker compounds were more fruity and less ashy. Among these two groups of wines, the low smoke impacted and non-impacted wines, the wines that had low smoke impact were less fruity compared to the non-impacted wines. This could be attributed to the fruity aromas masking the smoky aromas and vice versa, which was seen in previous studies where low levels of smoke reduced the perception of fruit attributes in a wine^7,17,36^. The low smoke-impacted wines also had lower “ashy” scored when compared to the high smoke-impacted wines. The concentration of the free and total (free + bound) VP were the main drivers of the smoke-related attributes and it was confirmed using the MFA (Fig. 4), mean sensory values (Table 6), the total VP (Table 4), and individual bound glycoside (Table 5) values.

VP glycosides are non-volatile precursors and odorless. However, glycosidic bonds are released through fermentation, during wine storage, and potentially in the mouth through bacteria or enzymatic hydrolysis^14^. These events can release VPs and give rise to the smoky related characters, in particular the “ashy” term^14^. Acid hydrolysis was used to cleave the acid labile compounds to give a quantitative measure of the total VP concentrations which include both the free VP and acid labile forms. Other research pointed to the individual bound forms as the main driver of smoke^19,37^. However, in these other studies a much larger set of phenolic glycosides were measured. This study showed that the amount of total VPs (Table 4) was reflective of the smoke impact seen in the wines (Tables 6 and 7) as seen with the high RV coefficients between the total VP and the DA which represents a good relation between the two data sets.

Across the free VP, total VP and individual bound glycosides, there were differences in the non-impacted samples across varietals such as between the Malbec and Syrah sample. However, there were no significant differences in the non-impacted Cabernet Sauvignon sample across different sites. This could indicate that baseline levels of the VPs and individual bound glycosides would be consistent across each varietal. When compared to the study done by Crews et al.^20^, the sum of glycosides was below 6 µg/L for baseline samples, which is consistent with the findings in this study for the non-impacted Cabernet Sauvignon tested. Syrah is a varietal that is known to have naturally elevated levels of VPs and the individual bound glycosides. These elevated values makes it significantly different from other non-impacted varietals. This was seen in the values for 21SY_L_NST being significantly different from 21MA_K_NST for the different VPs and individual bound glycosides (Tables 3, 4 and 5). It This shows that different varieties can have different baseline levels of VPs present in them naturally.

There was a difference between the “ashy” scores across different wine varietals and across locations for the same varietal. The variations could be attributed to concentration differences of free VPs, total VPs, and bound glycosides which vary naturally across different varietals and the amount of smoke exposure based on location as seen in Tables 1, 3 and 4, and 5^41–43^. Across all the wines evaluated, wines from Dry Creek Valley and St. Helena generally had the highest score for “ashy”. Within those locations, Cabernet Sauvignon wines had the highest score for “ashy”. Next when looking at the different varietals, wines made from Dry Creek Valley (20MA_D_ST and 20SY_I_ST) were highly smoke impacted and St. Helena (20CF_B_ST and 20PV_H_ST) were smoke-impacted at a medium level. It was observed that even with different varietals, the extent of smoke impact varied mainly by the location of the grapes as the location determined the amount of smoke exposure and VPs absorbed by the grapes. The varietal was a secondary effect. In Oakville where the smoke impact was relatively low, the “ashy” rating across varietals were similar ( 20CS_F_ST, 20_CF_C_ST, and 20ME_E_ST). The wines all had relatively low “ashy scores” due to lower grape smoke exposure compared to Dry Creek Valley and St. Helena. The wines from Dry Creek Valley and St. Helena also had the highest concentrations of free VPs, total VPs, and bound glycosides confirming that these marker compounds are driving smoke taint character in wine, confirming previous findings^12,21,41^. When comparing the smoke-exposed wines to their non-smoke counterparts, the Syrah (21SY_L_NST & 20SY_I_ST), Malbec (21MS_K_NST & 20MA_D_ST) and Cabernet Sauvignon (21CS_I_NST & 20CS_E_ST, 21CS_J_NST & 21CS_K_ST (intentional smoked)) wines, there was large differences in smoke marker compound levels which correlated with ashy scores and overall smoke impact. In comparing the smoke-exposed wines’ total VPs to some of the known threshold values guaiacol (23 µg/L), *m*-cresol (20 µg/L), *o*-cresol (62 µg/L), and *p*-cresol (64 µg/L) reported by Parker et al.^5^, some of the smoke-exposed wine has lower total VP values than the threshold values yet they are still perceived as smoky and ashy. This suggests a synergistic impact among VPs as discussed by McKay et al.^42^. Ultimately, with the data captured, it is not just the duration of fruit smoke exposure that is important but the proximity of the vineyard in relation to the smoke event as that determines the amount of VPs present in the smoke^43^. VPs in the gas phase have short lifetimes and break down within a few hours in the atmosphere^43^. It will be of great benefit if the released VPs in the smoke can be measured and related to grape and wine composition. However, this will most likely only be possible under controlled smoke situations as access to active wildfire zones are restricted^41^.

## Conclusion

The overall smoke impact on the wines was driven primarily by the location (origin) of the grapes, including proximity of the vineyard to the smoke event. The varietal impact of the grapes on smoke expression is small when compared to the location. Fresh smoke contains the most VPs and thus vineyards in close proximity to fires were exposed to more VPs in the atmosphere leading to more absorption. However, it is known that topography is another important factor to consider when determining risk from nearby fires^44^. Furthermore the direction and speed of air currents can have a large impact^45^. The wind could be blowing the smoke in the opposite direction from a vineyard, hence even in close proximity, these grapes may not be smoke impacted^46^. A modified descriptive analysis is a good rapid tool that can be used with minimal training to train a panel of relative experience to agree on a set of sensory terms to describe a product. However, additional studies are needed to determine smoke threshold levels in the different grape and wine matrixes both from a VP standpoint and a sensory standpoint.

## Electronic supplementary material

Below is the link to the electronic supplementary material.

Supplementary Material 1

## Data Availability

The datasets used and/or analysed during the current study is available from the corresponding author on request.
