# Supplementary material for: Using sensory and instrumental analysis to assess the impact of grape smoke exposure on different red wine varietals in California
Source: Sci Rep. 2024 Nov 7;14:27033. doi: 10.1038/s41598-024-77041-1 (PMC11541719; doi:10.1038/s41598-024-77041-1)
Supplement: Supplementary file 1 — Supplementary Material 1 [file 41598_2024_77041_MOESM1_ESM.pdf]

Using sensory and instrumental analysis to assess the impact of grape smoke exposure on different red wine varietals in California

Authors: Lik Xian Lim[1], Cristina Medina-Plaza[1], Ignacio Arías-Perez[1], Y. Wen[1], Bishnu Neupane[1], Larry Lerno[1], Jean-Xavier Guinard[2] and Anita Oberholster[1*]

[1]Department of Viticulture and Enology, University of California Davis, Davis, 95616, CA, USA
[2]Department of Food Science and Technology, University of California Davis, Davis, 95616, CA, USA
*Corresponding author

Supplementary Files

Supplementary Table S1: GC-MS/MS analysis parameters used for compound identification and quantification. RT- retention time, CE- collision energy

Compound Name	Precursor ion	MS1 res	Product ion	MS2 res	RT	Left Delta RT	Right Delta RT	Dwell (ms)	CE (V)
d-guaiacol	127.1	Wide	109	Wide	8.84	0.20	0.14	82.6	10
d-guaiacol	127.1	Wide	81	Wide	8.84	0.20	0.14	82.6	20
guaiacol	123.9	Wide	109	Wide	8.86	0.12	0.14	82.6	10
guaiacol	123.9	Wide	81	Wide	8.86	0.12	0.14	82.6	20
creosol	138.1	Wide	123	Wide	9.61	0.12	0.14	54.7	10
creosol	138.1	Wide	95	Wide	9.61	0.12	0.14	54.7	20
d-creosol	141.1	Wide	126	Wide	9.61	0.12	0.14	54.7	10
d-creosol	141.1	Wide	98	Wide	9.61	0.12	0.14	54.7	20
d-*o*-cresol	115	Wide	113	Wide	9.87	0.12	0.14	67.1	20
d-*o*-cresol	115	Wide	81	Wide	9.87	0.12	0.14	67.1	30
o-cresol	108	Wide	107	Wide	9.91	0.12	0.14	45.5	15
o-cresol	107	Wide	77	Wide	9.91	0.12	0.14	45.5	15
phenol	94	Wide	66	Wide	9.94	0.12	0.14	43.6	10
phenol	94	Wide	65	Wide	9.94	0.12	0.14	43.6	20
d-4-ethylguaiacol	157	Wide	139	Wide	10.15	0.12	0.14	42.4	10
d-4-ethylguaiacol	157	Wide	96	Wide	10.15	0.12	0.14	42.4	30
4-ethylguaiacol	151.8	Wide	137	Wide	10.19	0.12	0.14	73.3	10
4-ethylguaiacol	151.8	Wide	94	Wide	10.19	0.12	0.14	73.3	30
d-*pm*-cresol	115	Wide	113	Wide	10.54	0.12	0.14	67.1	20
d-*pm*-cresol	115	Wide	85	Wide	10.54	0.12	0.14	67.1	20
pm-cresol	108	Wide	107	Wide	10.58	0.12	0.14	48.5	15
pm-cresol	107	Wide	77	Wide	10.58	0.12	0.14	48.5	15
2,3-dimethoxyphenol	154	Wide	139	Wide	10.67	0.12	0.14	67.1	5
2,3-dimethoxyphenol	154	Wide	65	Wide	10.67	0.12	0.14	67.1	20
d-4-ethylphenol	126.1	Wide	111	Wide	11.29	0.12	0.14	54.7	10
d-4-ethylphenol	126.1	Wide	80	Wide	11.29	0.12	0.14	54.7	30
4-ethylphenol	121.9	Wide	107	Wide	11.30	0.12	0.14	82.6	10
4-ethylphenol	121.9	Wide	77	Wide	11.30	0.12	0.14	82.6	30
d-syringol	160	Wide	142	Wide	11.99	0.12	0.14	82.5	10
d-syringol	160	Wide	114	Wide	11.99	0.12	0.14	82.5	20
syringol	153.9	Wide	139	Wide	12.02	0.12	0.14	82.5	5
syringol	153.9	Wide	65	Wide	12.02	0.12	0.14	82.5	20
4-methylsyringol	168	Wide	153	Wide	12.71	0.12	0.14	110.4	5
4-methylsyringol	168	Wide	125	Wide	12.71	0.12	0.14	110.4	10

Supplementary Table S2: LC-MS/MS analysis parameters, multiple reaction monitoring was used for compound identification and quantification

Compound Name	Precursor Ion	Product Ion	Fragmentor (V)	Collision Energy (V)	Retention Time (min)	Retention Window	Polarity
Salicin	304	269	70	5	2.41	0.8	Positive
Salicin	304	107	70	10	2.41	0.8	Positive
Guaiacol gentiobioside	466	325	90	5	3.05	0.8	Positive
Guaiacol gentiobioside	466	163	90	10	3.05	0.8	Positive
Guaiacol gentiobioside	466	145	90	20	3.05	0.8	Positive
d3-Guaiacol gentiobioside	469	325	90	5	3.05	0.8	Positive
d3-Guaiacol gentiobioside	469	163	90	10	3.05	0.8	Positive
Phenol penthex	406	295	70	5	3.24	1	Positive
Phenol penthex	406	163	70	10	3.24	1	Positive
Phenol penthex	406	133	70	15	3.24	1	Positive
d3-Syringol gentiobioside	501	163	90	15	3.45	0.8	Positive
d3-Syringol gentiobioside	501	145	90	20	3.45	0.8	Positive
Syringol gentiobioside	496	325	90	5	3.5	0.8	Positive
Syringol gentiobioside	496	163	90	15	3.5	0.8	Positive
Syringol gentiobioside	496	145	90	20	3.5	0.8	Positive
Guaiacol glucoside	304	163	70	5	3.66	0.8	Positive
Guaiacol glucoside	304	145	70	5	3.66	0.8	Positive
Guaiacol glucoside	304	125	70	5	3.66	0.8	Positive
d5-Phenol rutinoside	425	309	90	5	3.69	0.8	Positive
d5-Phenol rutinoside	425	147	90	10	3.69	0.8	Positive
Phenol rutinoside	420	309	90	5	3.76	0.8	Positive
Phenol rutinoside	420	164	90	5	3.76	0.8	Positive
Phenol rutinoside	420	147	90	10	3.76	0.8	Positive
Amygdalin	475	325	80	5	3.89	0.8	Positive
Amygdalin	475	163	80	15	3.89	0.8	Positive
Guaiacol penthex	436	295	70	5	4.05	1	Positive
Guaiacol penthex	436	163	70	10	4.05	1	Positive
Guaiacol penthex	436	133	70	15	4.05	1	Positive
Syringol penthex	471	295	90	5	4.4	1	Positive
Syringol penthex	471	163	90	15	4.4	1	Positive
Syringol penthex	471	155	90	15	4.4	1	Positive
Cresol penthex	420.2	295	70	5	4.44	1	Positive
Cresol penthex	420.2	163	70	15	4.44	1	Positive
Cresol penthex	420.2	133	70	15	4.44	1	Positive
d3-Guaiacol rutinoside	453	309	90	5	4.54	0.8	Positive
d3-Guaiacol rutinoside	453	147	90	10	4.54	0.8	Positive
Guaiacol rutinoside	450	309	90	5	4.58	0.8	Positive
Guaiacol rutinoside	450	164	90	10	4.58	0.8	Positive
Guaiacol rutinoside	450	147	90	10	4.58	0.8	Positive
4-Methylsyringol gentiobioside	510	325	90	10	5.08	0.8	Positive
4-Methylsyringol gentiobioside	510	163	90	15	5.08	0.8	Positive
4-Methylsyringol gentiobioside	510	145	90	20	5.08	0.8	Positive
d5-Cresol rutinoside	439	309	90	5	5.56	0.8	Positive
d5-Cresol rutinoside	439	147	90	10	5.56	0.8	Positive
Cresol rutinoside	434	309	90	5	5.67	0.8	Positive
Cresol rutinoside	434	164	90	5	5.67	0.8	Positive
Cresol rutinoside	434	147	90	10	5.67	0.8	Positive
4-MS penthex	480	295	90	10	5.9	1	Positive
4-MS penthex	480	169	90	15	5.9	1	Positive
d3-4-Methylguaiacol rutinoside	467	309	90	5	6.28	0.8	Positive
d3-4-Methylguaiacol rutinoside	467	147	90	10	6.28	0.8	Positive
4-Methylguaiacol rutinoside	464	309	90	5	6.31	0.8	Positive
4-Methylguaiacol rutinoside	464	309	90	5	6.31	0.8	Positive
4-Methylguaiacol rutinoside	464	164	90	10	6.31	0.8	Positive
4-Methylguaiacol rutinoside	464	147	90	10	6.31	0.8	Positive

Supplemental Table S3: Descriptive analysis descriptors and preparation.

s/n	Category	Descriptor	**Recipe**	Brand
1	Aroma	Cigarette smoke	Pinch of ash of Camel Brand Loose Leaf Tobacco + 20mL Base wine	No. 91 Danish Export Virginia Blend Cigarete Tobacco.
2	Aroma	Musty	2cm square Wet Cardboard + 5mL Water to wet evenly	
3	Aroma	Medicinal / Brett	1 drop 40% Phenol Solution + 40mL Base Wine	
4	Aroma	Liquid Smoke	1mL Wrights Liquid smoke hickory with 40mL Base wine.	Wright's Liquid Smoke Concentrated Seasoning, Hickory
5	Aroma	Sweet BBQ	1mL Stubbs Mesquite Liquid Smoke with 40mL Base wine.	Stubbs Mesquite Liquid Smoke
6	Aroma	Menthol	1 ml solution (3 drops Eucalyptus oil in 100 ml water) + 40 ml Base wine	Nature's Truth Eucalyptus 100% pure essential oil
7	Aroma	Tar	1 slice Bicycle inner tube touch with Asphalt cement	Bell 12.5" Bicycle inner tube, Black Jack All weather Roof cement
8	Aroma	Solvent	0.125ml acetone, 1 drop ethyl acetate, 50ml Base wine	Acetone- Day logic brand 100% Acetone
9	Aroma	Red Fruit	2 Frozen Dark Cherry, 2 Frozen Raspberry, 1 Frozen Strawberry cut into cubes and smashed	**Strawberry**: Cascadian Farm (Sedro-Woolley, WA) **Dark Cherry & Raspberry:** Woodstock Organic
10	Aroma	Dark fruit	4 Frozen Blackberry, 2 Dark Frozen cherries, 5 Frozen Blueberry, all cut and smashed. + 1 drop Everclear	**Blueberry**: Cascadian Farm (Sedro-Woolley, WA). **Dark Cherry**: Woodstock Organic. **Blackberries:** Sthalbush Island Farms. **Everclear:** 120 proof
11	Aroma	Cooked fruit	1 tsp of each Blueberry jam, Blackberry jam, 1/2 tsp Strawberry and 1/2 Raspberry Jam in 20 mL Base wine	**Wild Blueberry Jam**- St Dalfour brand. **Strawberry, Blackberry, Raspberry Preserve**- Smuckers
12	Aroma	Dried fruit	1/8 Pcs Prune + 4 Raisins + 1/4 Dried Figs +10mL Base wine	**Prunes:** Sunsweet D'Noir Prunes preservative free. **Raisins:** Sunmaid raisins. **Figs:** Sun maid mission figs
13	Aroma	Bell pepper	0.5 cm square piece of Green bell pepper + 40ml Base wine, soak for 10 mins,	
14	Aroma	Spice	1 chip Nutmeg + 1 chip Cinnamon + 4 pcs All spice + 4pcs Cloves, in 40mL Base Wine Soak 45 mins and filter	**Nutmeg:** Morton and Barret, **Cinnamon, All spice, Cloves:** Mc Cormick
15	Aroma	Alcohol Hotness	16% Alcohol final content, 270mL/L 60% v/v Everclear	Everclear, 120 proof
16	Mouthfeel	Sweet	Low 5g/L Fructose, High 12.5g/L Fructose	Now Real Food
17	Mouthfeel	Sour	Low 0.5g/L Tartaric Acid, High 2g/L Tartaric Acid	
18	Mouthfeel	Bitter	Low- 500mg/L Caffeine, High 1000mg/L	Caffeine Anhydrous, VWR Life science
19	Mouthfeel	Dry	Low- 200mg/L Alum, High 620mg/L Alum	Mc Cormick
20	Mouthfeel	Hot	Low- 85mL/L 60% v/v Everclear, High- 170mL/L 60% v/v Everclear	Everclear, 120 proof
21	Mouthfeel	Viscosity	Low- plain Water, High 2g/L CMC in water	Sugarman candy.
22	Ashy	Ashy Aftertaste	Burnt Leeks ash in water, 100g leeks to 1000g water to make concentrate, Concentrate : Water, 1 :5 for 100% solution. ,	

#Base wine- where applicable , all aroma, taste, and mouthfeel standards made with Franzia Pinot Noir / Carmenere blend

Supplemental Table S4: Basic chemical composition of grapes used for winemaking.

Wine	Brix (°)	pH	TA (g/L)	Malic (mg/L)	NOPA (mg/L)	NH3 (mg/L)	YAN (mg/L)
DA 1							
20CS_A_ST	27.83 ± 0.45 C	3.68 ± 0.08 D	4.49 ± 0.29 D	2314 ± 262 D	80.67 ± 1.53 CD	35.00 ± 2.00 B	109.67 ± 3.21 D
20CS_B_ST	24.60 ± 0.17 H	3.94 ± 0.01 C	3.36 ± 0.04 G	2390 ± 53 D	64.00 ± 2.65 F	17.33 ± 4.51 E	78.33 ± 1.15 G
20CS_C_ST	25.37 ± 0.15 F	3.63 ± 0.03 DE	4.02 ± 0.09 E	1418 ± 67 G	66.67 ± 5.51 F	25.33 ± 0.58 D	88.00 ± 5.2 F
20CS_D_ST	26.03 ± 0.06 E	3.60 ± 0.02 DE	5.64 ± 0.13 B	3131 ± 49 B	74.67 ± 2.08 E	34.67 ± 4.51 BC	100.00 ± 5.29 E
20CS_E_ST	23.95 ± 0.07 I	3.43 ± 0.01 FG	5.98 ± 0.08 A	3778 ± 14 A	76.50 ± 3.54 DE	38.00 ± 1.41 B	108.00 ± 4.24 D
20CS_F_ST	28.00 ± 0.00 C	3.55 ± 0.01 EF	5.29 ± 0.36 C	2739 ± 124 C	90.33 ± 5.86 B	36.67 ± 2.08 B	120.67 ± 5.03 B
20CS_G_ST	26.90 ± 0.10 D	3.91 ± 0.01 C	3.62 ± 0.02 FG	1986 ± 130 E	101.00 ± 3.61 A	34.67 ± 3.21 BC	130.00 ± 6.24 A
20CS_H_ST	25.83 ± 0.06 E	3.71 ± 0.02 D	4.60 ± 0.08 D	79 ± 3 H	45.67 ± 4.04 G	116.33 ± 1.15 A	116.00 ± 0.00 BC
21CS_I_NST	24.97 ± 0.06 G	3.34 ± 0.03 G	5.76 ± 0.17 AB	2997 ± 62 B	36.00 ± 0.00 H	21.00 ± 1.00 DE	53.00 ± 1.00 H
21CS_J_NST	31.70 ± 0.17 B	24.80 ± 0.17 B	3.86 ± 0.01 EF	1490 ± 24 G	68.67 ± 0.58 F	19.67 ± 1.53 E	84.67 ± 1.15 F
21CS_K_ST	33.70 ± 0.26 A	26.07 ± 0.15 A	3.89 ± 0.01 EF	1699 ± 20 F	86.00 ± 0.00 BC	30.33 ± 2.08 C	110.67 ± 1.53 CD
DA 2							
20CF_A_ST	25.63 ± 0.06 E	3.76 ± 0.02 D	3.93 ± 0.13 EF	1679 ± 24.66 G	74.33 ± 1.53 E	22.33 ± 5.13 DEF	92.67 ± 2.89 FG
20CF_B_ST	26.27 ± 0.21 D	3.96 ± 0.01 B	3.25 ± 0.09 H	1463 ± 26.41 H	48.33 ± 2.52 G	14.33 ± 1.53 EF	60.33 ± 3.51 H
20CF_C_ST	27.40 ± 0.10 C	3.65 ± 0.07 F	3.68 ± 0.63 FG	1851 ± 58.43 F	78.67 ± 2.31 E	41.00 ± 18.25 ABC	112.67 ± 15.89 DE
20MA_D_ST	24.93 ± 0.06 H	3.87 ± 0.02 C	4.25 ± 0.03 CDE	3276 ± 74.06 A	95.67 ± 5.69 D	34.67 ± 2.52 BCD	124.67 ± 6.51 CD
20ME_E_ST	27.77 ± 0.31 B	3.44 ± 0.03 H	4.80 ± 0.34 B	1843 ± 47.79 F	93.33 ± 3.21 D	43.00 ± 6.24 ABC	129.00 ± 8.00 C
20ME_F_ST	25.27 ± 0.12 FG	4.04 ± 0.00 A	2.70 ± 0.00 I	1682 ± 21.46 G	76.00 ± 4.36 E	8.67 ± 2.08 F	83.33 ± 2.89 G
20PV_G_ST	25.67 ± 0.25 E	3.91 ± 0.01 BC	4.03 ± 0.08 DEF	2674 ± 24.01 C	130.33 ± 4.93 B	32.00 ± 2.00 CD	157.00 ± 3.61 B
20PV_H_ST	25.43 ± 0.12 EF	3.94 ± 0.01 B	3.46 ± 0.06 GH	2129 ± 41.40 D	74.33 ± 6.66 E	12.33 ± 4.62 EF	81.00 ± 2.65 G
20SY_I_ST	28.63 ± 0.29 A	3.70 ± 0.02 E	5.71 ± 0.15 A	3059 ± 55.56 B	137.67 ± 1.15 A	50.67 ± 8.02 A	180.00 ± 5.57 A
20ZN_J_ST	22.70 ± 0.10 J	3.48 ± 0.02 G	4.57 ± 0.07 BC	2158 ± 68.08 D	66.00 ± 3.00 F	25.67 ± 2.31 DE	87.00 ± 4.58 FG
21MA_K_NST	25.03 ± 0.12 GH	3.67 ± 0.01 EF	4.31 ± 0.03 CD	2008 ± 12.12 E	62.67 ± 1.53 F	47.00 ± 13.00 AB	101.33 ± 11.02 EF
21SY_L_NST	23.50 ± 0.10 I	3.49 ± 0.02 G	5.70 ± 0.04 A	3023 ± 12.50 B	115.00 ± 2.08 C	47.67 ± 13.28 AB	137.67 ± 18.82 C

Fisher’s LSD was used to determine differences between each column for each DA across wines
